# Disentangling the microRNA regulatory *milieu* in multiple myeloma: integrative genomics analysis outlines mixed miRNA-TF circuits and pathway-derived networks modulated in t(4;14) patients

**DOI:** 10.18632/oncotarget.6151

**Published:** 2015-10-19

**Authors:** Enrica Calura, Andrea Bisognin, Martina Manzoni, Katia Todoerti, Elisa Taiana, Gabriele Sales, Gareth J. Morgan, Giovanni Tonon, Nicola Amodio, Pierfrancesco Tassone, Antonino Neri, Luca Agnelli, Chiara Romualdi, Stefania Bortoluzzi

**Affiliations:** ^1^ Department of Biology, University of Padua, Padua, Italy; ^2^ Department of Molecular Medicine, University of Padua, Padua, Italy; ^3^ Department of Clinical Sciences and Community Health, University of Milan, and Hematology Unit, Fondazione IRCCS Ca' Granda Ospedale Maggiore Policlinico, Milan, Italy; ^4^ Laboratory of Pre-Clinical and Translational Research, IRCCS-CROB, Referral Cancer Center of Basilicata, Rionero in Vulture, Italy; ^5^ Myeloma Institute, University of Arkansas for Medical Sciences, Little Rock, AR, USA; ^6^ Functional Genomics of Cancer Unit, Division of Experimental Oncology, San Raffaele Scientific Institute, Milan, Italy; ^7^ Department of Experimental and Clinical Medicine, University of Study Magna Graecia, Catanzaro, Italy

**Keywords:** multiple myeloma, transciptional regulatory network, t(4;14) translocation, microRNA, expression profiling

## Abstract

The identification of overexpressed miRNAs in multiple myeloma (MM) has progressively added a further level of complexity to MM biology. miRNA and gene expression profiles of two large representative MM datasets, available from retrospective and prospective series and encompassing a total of 249 patients at diagnosis, were analyzed by means of i*n silico* integrative genomics methods, based on *MAGIA*^2^ and *Micrographite* computational procedures. We first identified relevant miRNA/transcription factors/target gene regulation circuits in the disease and linked them to biological processes. Members of the *miR-99b/let-7e/miR-125a* cluster, or of its paralog, upregulated in t(4;14), were connected with the specific transcription factors *PBX1* and *CEBPA* and several target genes. These results were validated in two additional independent plasma cell tumor datasets. Then, we reconstructed a non-redundant miRNA-gene regulatory network in MM, linking miRNAs, such as *let-7g, miR-19a, mirR-20a, mir-21, miR-29* family, *miR-34* family, *miR-125b, miR-155, miR-221* to pathways associated with MM subtypes, in particular the ErbB, the Hippo, and the Acute myeloid leukemia associated pathways.

## INTRODUCTION

Consolidated evidences indicate that microRNAs (miRNA) could be markedly modulated in human cancers, which leads them to be currently considered both emerging therapeutic targets and innovative intervention tools [1]. The oncogenic role or tumor suppressor activity of a number of miRNAs has been experimentally demonstrated in various tumors, including hematological malignancies [2-6]. In multiple myeloma (MM), as well, specific miRNAs have been identified as deregulated in distinct subgroups of patients mainly in association with IGH@ translocations or allelic imbalances, suggesting that individual miRNAs may play an important role in neoplastic transformation and progression of the disease [7-12]. MM is genomically unstable and broadly stratified on the basis of ploidy status: hyperdiploidy occurs as a primary event in approximately half of MM tumors that have a generally better prognosis, whereas non-hyperdiploid tumors are enriched in primary IGH@ translocations events. Of these, the most prevalent are t(11;14) and t(4;14), which cause the deregulation of *CCND1* and *WHSC1/FGFR3* genes, respectively. Other genetic abnormalities arise during the evolution of the disease (e.g. p53 inactivation and/or deletion, Myc deregulation), and are specifically associated with the more advanced stages, such as extramedullary disease and plasma cell leukemia (PCL). This latter form of plasma cell dyscrasia, in particular, may occur *de-novo,* as primary event (pPCL), or derive as secondary evolution (sPCL) from primary MM tumor.

In previous investigations [7, 13], we have demonstrated that the main molecular prognostic groups in MM were characterized by the specific overexpression of miRNA or miRNA clusters, as in the case of *miR-99b/let-7e/miR-125a* in t(4;14) positive patients. In the same reports, we focused on the inference of targets of a few miRNAs differentially expressed among MM classes using a relatively simple method based on the anticorrelation of miRNA predicted targets, which highlighted a number of putative transcriptional relationships. The t(4;14) translocation is commonly considered as early unfavorable prognostic factor [14], but we are far from fully understanding its involvement in the disease. Evidences have also emerged indicating that clinical and molecular heterogeneity within this subgroup of MM patients could be present, which might also be associated with miRNA expression [15-17]. Finally, in a recent study involving a large and prospective cohort, we demonstrated that a minimal miRNA-based classifier model (including miR-17 and miR-886) is capable of improving risk stratification in MM [13].

Herein, we take advantage of genomic analyses applied to two independent sizeable and representative datasets, to generate a transcriptional and post-transcriptional regulatory networks modulated in MM, in order to define microRNAs impacting in regulatory circuits with potential functional and clinical relevance.

## RESULTS

In this study, we first considered two large independent MM datasets, one retrospective, newly obtained by our group (“NewMM96”), and one prospective, already available (“MyIX153”), encompassing, respectively, 96 and 153 patients at diagnosis. Table 1 describes patient data, for each dataset.

**Table 1:** Summary of MM patients' data and cytogenetic features. P-value indicates the result of Fisher's exact test of independence between patient classes and sample distribution

Description	MyIX153	NewMM96	P-value
Sex			
M	88 (57.5%)	48 (50%)	0.29
F	65 (42.5%)	48 (50%)	
Age			
≤ 70	109 (71.2%)	63 (65.5%)	0.39
> 70	44 (28.8%)	33 (34.5%)	
del(13q)			
+	56 (36.6%)	50 (52%)	0.06
-	87 (56.9%)	46 (48%)	
n.d.	10	-	
t(4;14)			
+	22 (14.4%)	13 (13.5%)	0.85
-	121 (79.1%)	83 (86.5%)	
n.d.	10	-	
t(11;14)	22 (14.4%)	22 (22.9%)	0.17
+	121 (79.1%)	74 (77.1%)	
-	10	-	
n.d.			
t(14;16)			
+	4 (2.6%)	4 (4.2%)	0.72
-	139 (90.8%)	92 (95.8%)	
n.d.	10	-	
t(14;20)			
+	2 (1.3%)	1 (1%)	1
-	141 (92.2%)	95 (99%)	
n.d.	10	-	
Hyperdiploidy			
+	83 (54.2%)	32 (33.5%)	0.003
-	60 (39.2%)	54 (56%)	
n.d.	10	10	
1q+			
+	56 (36.6%)	41 (42.7%)	0.22
-	87 (56.9%)	45 (46.9%)	
n.d.	10	8	
del(1p)			
+	25 (16.3%)	6 (6%)	0.1
-	118 (77.1%)	67 (70%)	
n.d.	10	22	

**Figure 1:**
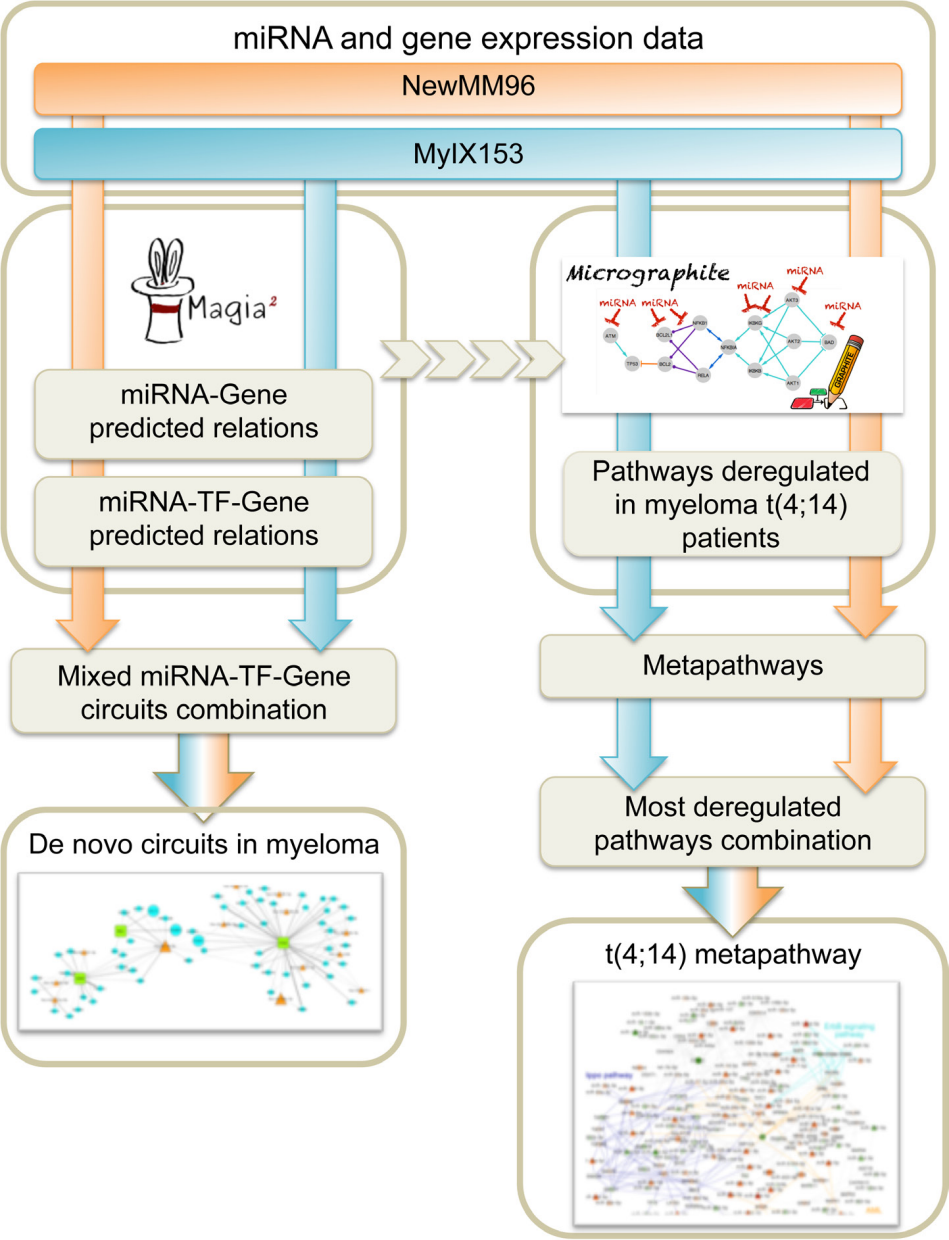
Summary of the computational workflow

We aimed at detecting most significant transcriptional and post-transcriptional regulatory networks modulated in MM, in order to define microRNAs impacting in regulatory circuits with potential functional and clinical relevance. The meta-analysis of the two miRNA and gene expression datasets were performed with a composite pipeline (Figure 1) designed to extract information from sequence and expression data, exploiting both an “*ab initio*” and a “*knowledge-based*” approach. The results of the two methods are complementary; the “*ab initio*” approach focusing on the discovery of new relations, while the other selecting the most involved relations among those described in biological pathways. The integrated strategy allowed us: (i) to first identify transcriptional and post-transcriptional regulatory networks in MM; then (ii) to reconstruct an informative and non-redundant miRNA-gene regulatory network in MM, linked to gene functions and known pathways; and finally (iii) to identify the most relevant pathways associated with MM subtypes.

### Ab intio reconstruction of miRNA/TF/gene transcriptional network

The first step of the pipeline is based on MAGIA^2^ method [18], which takes account of miRNAs and transcription factors (TF) interplay and allows identifying two types of mixed miRNA/TF/gene circuits, namely those describing (i) a TF that activates both a miRNA and its target gene and (ii) a miRNA that inhibits both a TF and its regulated gene.

**Figure 2:**
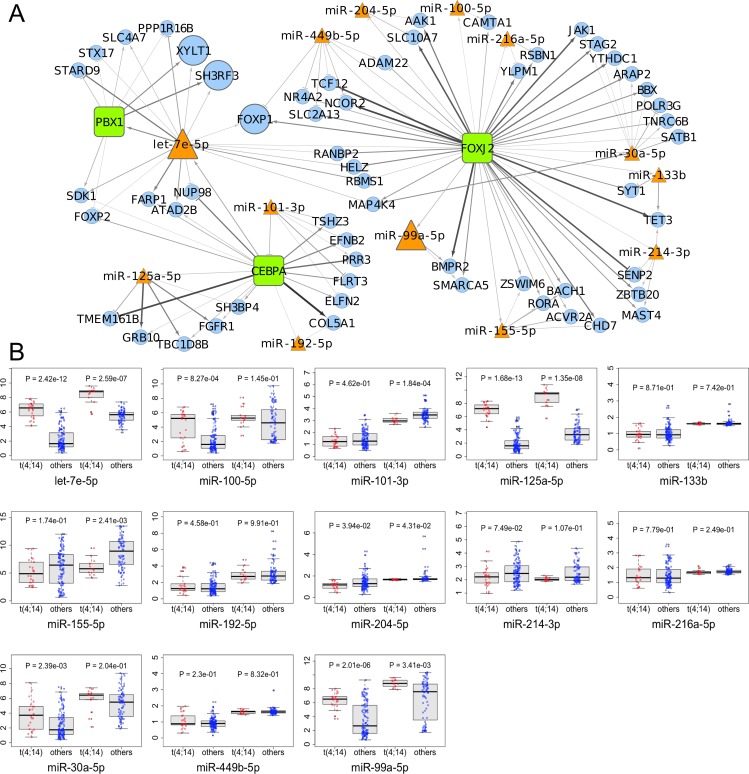
Transcriptional and post-transcriptional regulatory circuits in MM **A.** The network shows the eight nodes (bold-outlined larger shapes) included in relationships common to the networks obtained analyzing NewMM96 and MyIX153 datasets in parallel. Orange triangles represent microRNAs, green boxes Transcription Factors and light-blue circles the other coding mRNAs, while edges represent in-silico inferred relationships, with arrows and T-shaped edges showing respectively positive and negative correlations. Color intensities and edge widths are proportional to absolute correlation measures (where the relationship occurred in both datasets, the measure from the NewMM96 was chosen for edge attributes visualization). **B.** Boxplots show the expression levels of the miRNAs included in the network in the MyIX153 and the NewMM96 dataset. Red dots refer to t(4;14) patients, while blue ones represent non-t(4;14) patients expressions.

**Figure 3:**
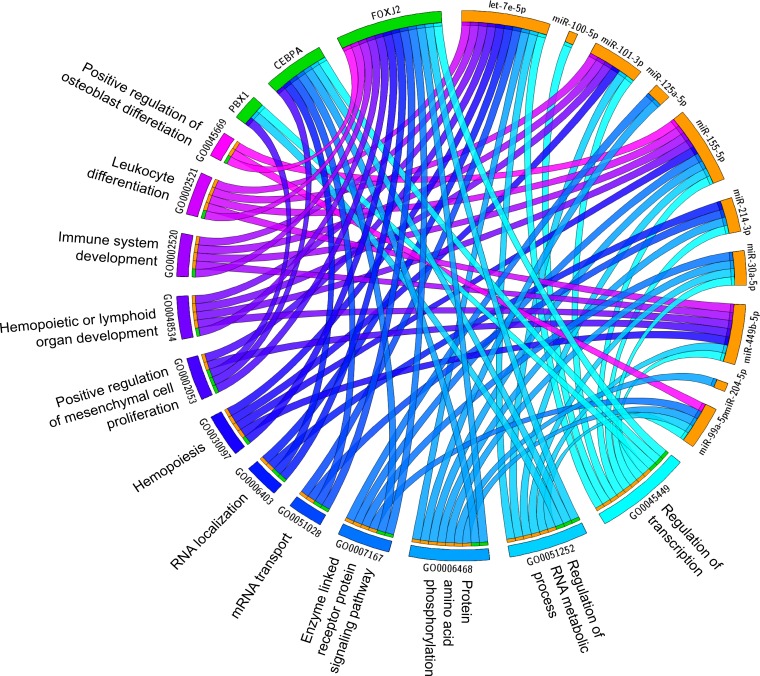
Impact of transcriptional and post-transcriptional regulators on biological process in MM The Circus plot shows the correspondence between miRNAs and TFs included in the network of Figure 2A and the main functional categories (Gene Ontology Biological Processes) to which their target genes belong.

MAGIA^2^ analysis identified 139 and 81 mixed miRNA/TF/gene circuits (Supplementary Table 1), respectively, from the analysis of the NewMM96 and of the MyIX153 datasets. Among circuits detected by the two parallel analyses, to strengthen the analysis and prevent that results might be affected by batch- or cohort-specific effects, we focused on the interlaced regulatory triplets that were commonly identified in both datasets: two most relevant overlaping circuits have been identified that involved the members of the *miR-99b/let-7e/miR-125a* cluster on chromosome 19 (or of its paralog on chromosome 21), which have been demonstrated as specifically upregulated in t(4;14) [7, 13], the pre-B-cell leukemia homeobox 1 (*PBX1*) transcription factor, and the *SH3RF3* and *XYLT1* genes. These are linked with the *CEBPA*/let-7e relation in both datasets, but coupled with different target genes: *FARP1* (in NewMM96) and *NUP98* (in MyIX153). This observation gives a hint of the two-fold advantage of the parallel analysis of two datasets: not only the identification of common and strong elements, but also the integration and complementation of dataset-specific results, which ultimately provide a broader picture of the disease-associated circuits, as previously demonstrated [19-21]. To prevent that bridges among circuits might be masked by the occurrence of marginally significant correlations (concordant but not identified in both dataset based on the defined correlation thresholds), the results from the two MAGIA^2^ analyses were merged and the nodes sharing relationships in both datasets were selected: as shown in Figure 2A, a new “child” network have been finally derived that included such eight nodes along with their first neighbors (for a total of 13 miRNAs and 60 genes) in the mixed circuits network. Figure 2B shows the expression levels of the miRNAs included in the networks of Figure 2A in t(4;14)-positive and -negative patients, respectively in the MyIX153 and in the NewMM96 dataset. Expression level of the transcripts included in the mixed network, in the two considered sample sets, are shown in Supplementary Figure 1. Moreover, we investigated if miRNAs and TFs included in the Figure 2A network tend to regulate genes associated to specific functional categories. The Circos plot in Figure 3 provides a summary of the main functional categories (GO Biological Processes) in which the genes identified in the circuits in Figure 2A are annotated: specifically, it highlights the correspondence between miRNAs/TFs and the functional categories to which the connected genes belong.

To strengthen the reliability of the identified connections, we ran MAGIA^2^ analysis under the same computational criteria in two independent, publicly available, plasma cell tumor datasets. The first one (“MM60”) included 60 MM tumors at diagnosis [9], whereas the second dataset (“PCL29”) included 29 patients with primary or secondary PCL. Notably, the analysis of the two validation sets confirmed the relationships involving the miRNA cluster miR-99b/let-7e/miR-125a with *PBX1* (*r* = 0.24 in MM60) and *CEBPA* (*r* = 0.49 in PCL29) TFs. It is worth noting that the associations were retained in the PCL dataset, namely in the most aggressive form among the plasma cell dyscrasias, suggesting that this transcriptional circuit could be identified in tumor plasma cells independently of the disease presentation.

The relevance of such findings described in the mixed network, involving the *miR-99b/let-7e/miR-125a* cluster overexpressed in MM patients with t(4;14), prompted us to further investigate, through a robust *in silico* approach, at what extent such cluster or other miRNAs might be involved in the biology of t(4;14) tumor itself.

### Characterization of t(4;14)-associated pathways

A topological pathway-based analysis has been therefore used to disentangle the t(4;14)-associated network. This approach, called *Micrographite* [22], exploits the *a priori* gene-gene and miRNA-gene relations described in pathways and in literature, in order to unravel relevant circuits specifically modulated, directly or indirectly, in samples stratified according to investigated parameters. The procedure, herein, has been applied considering miRNA and gene expression data of NewMM96 and of the MyIX153 datasets, both stratified according to the occurrence of t(4;14). Thus, contrasting positive and negative t(4;14) patients for each dataset, M*icrographite* analysis identified the most significantly modulated pathways for each dataset.

The analysis of the NewMM96 dataset led to the identification of 66 significant pathways, whereas 47 emerged in MyIX153; of these, 17 were commonly detected (26% and 36% of the identified pathways in the two datasets, respectively; Supplementary Table 2). This is a relevant overlap, considering the different generation arrays used in original studies and the intrinsic cohort-specific differences.

**Figure 4:**
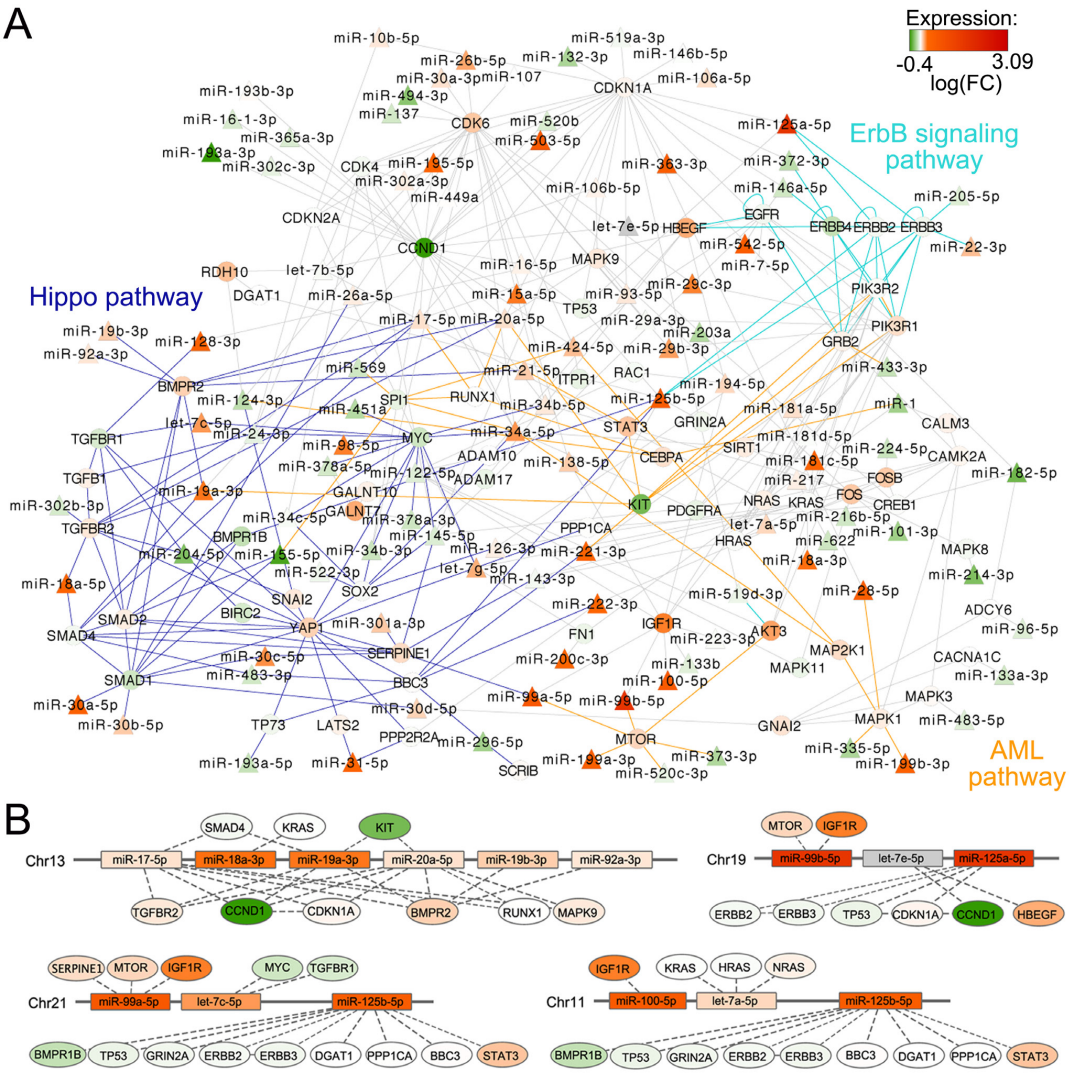
Union of KEGG path-derived networks associated to t(4;14) phenotype by micrographite analysis of NewMM96 and MyIX153 datasets **A.** Union network of the two meta-pathways build on the two and four most significant paths associated with t(4;14) phenotype in MyIX153 and NewMM96 datasets, respectively. For reader's convenience, only miRNAs and first neighbors are visualized, whereas the complete network is depicted in Supplementary Figure 2. miRNAs are represented with triangles, genes with circles. Cyan, blue and orange solid edges connected genes included in ErbB signaling, Hippo and acute myeloid leukemia pathways, respectively. The color scale bar (bottom right) is referred to the fill-in color of each node, representing the log2ratio between t(4;14) and non-t(4;14) patients mean expression levels. Gray nodes represent elements whose expression levels, measured in the MyIX153 and NewMM96 dataset, presented a minimal discrepancy, not exceeding ± 0.1. The 23 transcripts that exceeded ± 0.1 threshold were discarded from the network. **B.** Sketch of the genomic structure of four miRNA clusters represented in the network. For each miRNA in a cluster, target genes are shown as connected node; miRNA/gene color indicates the log2ratio between t(4;14) and non-t(4;14) patients mean expression levels, as is in the network of panel A.

A single gene can be included in many pathways, and different pathways can be highly connected and share genes. Moreover, only a portion of the pathways can be modulated in a specific condition, as the occurrence of the t(4;14) on which we focused. Thus, exploiting pathway overlaps, *Micrographite* has the ability to dissect the obtained results highlighting only the most important set of interactions inside a pathway. Here, aimed at providing a better and a more complete picture of regulatory mechanisms in the disease, the results from the two datasets were combined. Specifically, the genes and microRNAs included in the upper most significant 10^th^ percentile of the identified pathways were selected for each dataset (which corresponded to the genes and miRNAs included in the two and four most significant pathways in MyIX153 and NewMM96 datasets, respectively; Supplementary Table 2) and used to build a comprehensive meta-pathway. Finally, the obtained meta-pathway has been re-analyzed to rank the portions associated mostly with t(4;14) translocation. Figure 4 shows the union network, ultimately giving a non-redundant, functionally informative data-driven picture. For reader's convenience, only microRNAs and the first nearest neighbors (namely, the primary connections) have been visualized, whereas the whole network is reported in Supplementary Figure 2.

Of note, of the 17 pathways common to both datasets after the first step of the analysis, seven were still represented in the final union network. Three of seven were of major interest in the context of MM: namely, the ErbB pathway, the Hippo pathway, and the acute myeloid leukemia associated pathway. Interestingly, in the final network several genes, also largely described in MM biology, emerged as interconnected nodes within these pathways, and specific miRNAs (most of which already known to be involved in MM biology) represent the connection between them: *let-7g, miR-19a, mirR-20a, mir-21, miR-29* family, *miR-34* family, *miR-125b, miR-155, miR-221*. We also found that *CCND1* and the related *CDK* genes network are putatively associated with a large number of miRNA species, among which there was the *miR-125a*, a marker of t(4;14) translocation.

Based on the availability of clinical information in MyIX153 dataset, we tested whether the differential expression of any of these miRNA may somehow be associated with clinical outcome in t(4;14), namely if their upregulation might hypothetically confer prognostic (dis)advantage in terms of OS or PFS. Although the limited number of cases prevents the possibility to trace definitive conclusions, the results of the analysis indicated that the overexpression of members of *miR-17∼92* cluster could be related to increased risk of death (Supplementary Table 3A and Supplementary Figure 3A), whereas the overexpression of *miR-520c-3p* could be associated with lower risk of early progression (Supplementary Table 3B and Supplementary Figure 3B). However, none of the miRNAs retained significance after correcting for multiple test, which demands for cohorts with higher number of t(4;14) patients to confirm this preliminary finding.

## DISCUSSION

Herein, we have taken virtue of state-of-the-art computational procedure to provide a comprehensive and biologically relevant view of the transcriptional regulatory networks in MM, highlighting microRNAs with potential functional significance and clinical relevance. The present study overcomes most of the limitations of our previous report [7], which had been focused on the inference of targets of a few miRNAs found differentially expressed among MM classes: (i) implemented procedures, run simultaneously on two different large datasets of MM at diagnosis and validated in two additional independent datasets; (ii) a refined method for inference of mixed miRNA-TFs-target circuits from expression data; (iii) updated target predictions and annotations; and (iv) a topology based methods considering miRNAs and gene expression variations in pathway-derived networks. All together, these aspects represented a substantial improvement to unravel subtle, but potentially biologically meaningful, variations in the expression of miRNA/TFs/genes. We have recently demonstrated the advantages of such novel integrative genomic approaches in disclosing the connection between the differential miRNome and transcriptome in hyperdiploid *versus* non-hyperdiploid MM tumors [23].

Undoubtedly, the major finding involved the miRNA cluster *miR-99b/let-7e/miR-125a*, which represents so far one of the most promising field of study in MM given the exclusive association with the t(4;14) translocation [7, 13]. So far, little is known about the TFs involved in t(4;14)-associated circuits in myeloma. *PBX1* has been largely described in acute lymphoblastic leukemia; notably, it has been suggested that, in complex with *MEIS2, PBX1* is involved in transcriptional regulation mediated by *KLF4*, which has been previously shown by us as specifically overexpressed in t(4;14) patients [24, 25]. Upregulation of *CEBPA* has been linked to favorable prognosis in both adult and pediatric acute myeloid leukemia patients. Based on the close relationship that binds the overexpression of *miR-99b/let-7e/miR-125a* and t(4;14), we may speculate that the modulation of *PBX1* and *CEBPA,* excluding for obvious reasons the direct relationship with the translocation event, could be somehow related to t(4;14) beyond the *WHSC1*/*FGFR3* deregulation mechanism and in tight connection with the overexpression of such miRNA cluster.

Therefore, prompted by this hypothesis, in the second part of our study we focused on the reconstruction of the topological pathways associated with t(4;14) in MM, leading to the identification of main interlaced pathways. The first two pathways that are worth mentioning are associated (i) with the ErbB receptor signaling and (ii) with genes (*c-KIT, STAT3, AKT3*) that have been mainly described as increased in acute myeloid leukemia, in which the crucial role of *CEBPA* has been widely demonstrated [26, 27]. A bridge could be established by the PI3K/AKT/mTOR1 pathway, that has been demonstrated to direct the lineage fate during myelopoiesis at least in part by controlling the phosphorylation of *CEBPA* [28]. The transcriptional network recognized a putative functional association interposing miR-125a between *ERBB2/ERBB3, TP53* and *CDKN1A*. The relationship between miR-125a and *ERBB* genes family has indeed been demonstrated in several tumors [29], whereas the induction of a p53-dependent tumor suppression specifically induced by miR-125a inhibition has been recently described by us in MM [30]. A parallel connection has been identified between miR-29 family and *CDK6*, interaction on which reside the miR-29-antiproliferative effects reported in B-cell lymphomas [31].

Furthermore, the topological map highlighted the modulation and the involvement of the Hippo pathway. Recently, it has been described that low levels of *YAP1*, a co-activator of the Hippo pathway under the control of the serine-threonine kinase STK4, prevents the ABL1-induced p53-independent apoptosis stimulated by DNA-damage in MM tumor cells [32]. Significantly higher *YAP1* levels have been identified associated with the large fraction of t(4;14) cases; this was more evident in MyIX153 dataset (*P* = 0.00065, Supplementary Figure 4A) although a concordant trend was also observed in NewMM96 (Supplementary Figure 4B). Such higher *YAP1* levels could be in apparent contrast with what expected in cases presenting the t(4;14) translocation, which is commonly considered as unfavorable in MM; however, this might partially be explained with the known heterogeneity of t(4;14) patients, that are stratified in two prognostically distinct entities [14-17]. In line with this consideration, t(4;14) with higher *YAP1* levels in MyIX153 presented a slightly, although not significantly, more favorable outcome than other cases (Supplementary Figure 5). The Hippo network has been connected with miRNAs that resulted consistently, albeit faintly, modulated in t(4;14). These included the well-known *miR-17∼92* cluster, whose miRNAs were linked with members of the TGFβ-associated Ser/Thr-kinases pathway, such as *TGFBR2, BMPR2* and the signal transducer *SMAD4*. The involvement of these correlated genes in bone formation [33], together with the documented lower occurrence of osteolytic bone lesions in t(4;14) patients [34], hint that regulatory mechanisms involving *miR-17∼92* cluster and the TGFβ/Hippo pathways are worthy of further investigations.

## MATERIALS AND METHODS

### Datasets

#### Proprietary dataset (“NewMM96”)

Samples. Bone marrow aspirates from newly-diagnosed 96 MM patients were obtained during standard diagnostic procedures at the IRCCS Institution in Milan. A fraction of these samples (40 cases), whose expression had been also analyzed on old-generation array platforms, were described in previous report [7]. All patients gave their informed consent for molecular analyses. PCs were purified using CD138 immunomagnetic microbeads (MidiMACS, Miltenyi Biotec, Auburn, CA). The purity of the positively selected PCs (≥90%) was assessed by means of flow cytometry. All MM cases were investigated by fluorescence in-situ hybridization (FISH) for the major Immunoglobulin Heavy-Chain locus (IGH@) translocations and genetic lesions [13q14 deletion, TP53 deletion, gain of chromosome 1q21.3 (CKS1B) and deletion of 1p33 (CDKN2C)] using appropriate BAC clones (selected through UCSC Genome Browser at http://genome.ucsc.edu/) according to previously described procedures [35].

MiRNA and gene expression profiling. The total RNA extraction and quality assessment were performed as previously described [7]. Samples were profiled in accordance with the manufacturer's instructions on GeneChip^®^ miRNA 3.0 arrays. Raw data were extracted from CEL files and then normalized using robust multi-array average (RMA) procedure in the *affy* package for Bioconductor and the miRbase Release 18 annotations (www.mirbase.org) included in the corresponding *cdf* definition files available at the University of Michigan Brainarray portal (http://brainarray.mbni.med.umich.edu/Brainarray/Database/CustomCDF/18.0.0/version.html). Annotations were then updated to miRBase Release 20 definition using the *mirna.diff* files available at the miRbase website. The raw and normalized miRNA data are available through GEO accession number GSE70254.

Whole gene transcriptional profiles were then generated using GeneChip^®^ Gene 1.0 ST Array (Affymetrix Inc., Santa Clara, CA). Preparation of DNA single-stranded sense target, hybridization and scanning of the arrays (7G Scanner, Affymetrix Inc.) were performed according to the manufacturer's protocols. Log_2_-transformed expression values were extracted from CEL files and normalized using RMA procedure in the *affy* package for Bioconductor and the Transcript Cluster Annotations included in the *cdf* definition files version 18 available at the Brainarray portal.

#### MRC myeloma IX dataset (“MyIX153”)

The gene and microRNA expression profiles of one-hundred and fifty-three patients included in MRC Myeloma IX trial and described in our previous report [13] were considered for the present study. The whole expression data were publicly available at the Gene Expression Omnibus (GEO) repository under accession number GSE15695 and GSE41276 and were processed as previously described [13], using the Brainarray *cdf* definition files version 18, as done for the proprietary data.

#### GSE16558 dataset (“MM60”)

The gene and microRNA expression profiles of sixty MM patients publicly available under GEO accession GSE16558 were considered as validation set for the present study. The gene expression data, generated on GeneChip^®^ Gene 1.0 ST Array (Affymetrix Inc., Santa Clara, CA), were processed as described above for proprietary dataset, using the Brainarray cdf definition files version 18, as done for the proprietary data. The miRNA expression data, generated on Applied Biosystems Human TaqMan^®^ Low Density Array, were dowloaded as originally released by the Authors [9] and then reannotated to the updated miRBase Release 20 definition using the *mirna.diff* files available at the miRbase website.

#### Plasma cell leukemia dataset (“PCL29”)

The gene and microRNA expression profiles of twenty-nine plasma cell leukemia patients were considered as validation set for the present study. This cohort includes primary (pPCL) and secondary (sPCL) cases, the former described in previous studies from our group [36, 37]. The gene expression data, generated on GeneChip^®^ Gene 1.0 ST Array (Affymetrix Inc., Santa Clara, CA), and the miRNA expression data, generated on GeneChip^®^ miRNA 3.0 arrays, were processed as described above for proprietary dataset. The raw and normalized RNA data are available through GEO accession numbers GSE73452 and GSE73454.

### MAGIA^2^ analysis for the identification of mixed circuits involving miRNA/TF/mRNA

For each dataset, miRNA and transcripts expression data were analyzed using MAGIA^2^, to identify mixed circuits (triplets) involving miRNA/gene/transcription factor (TF; http://gencomp.bio.unipd.it/magia^2^/), as previously described [18]. Specifically, Targetscan was used as target prediction algorithm, and Pearson coefficient was used to measure relationships between microRNA and target mRNA expression profiles. Only the most variable 75% genes according to the coefficient of variation were considered. Lower threshold for absolute correlation coefficients within circuits was set to 0.2; 0.4 was used for miRNA/target binary relationships. Functional annotation analysis was performed using the standard procedure in the Database for Annotation, Visualization and Integrated Discovery (DAVID) tool [38], version 6.7. Circos plot [39] was generated using the online tool at http://mkweb.bcgsc.ca/tableviewer/.

### Micrographite analysis to detect most modulated pathway-derived networks

*Micrographite* pipeline allows integrating pathway topologies with predicted and validated miRNA-target interactions, to perform integrated analyses of miRNA and gene expression profiles, for the identification of modulated regulatory circuits involved in the disease in terms of both expression variations and differential strength of inferred interactions [22]. *Micrographite* has two steps: i) the extension of pathway annotation using miRNA-target interaction and ii) recursive topological pathway analysis on these networks. We considered network topologies derived from KEGG database by *Graphite* package [40] and miRNA-target gene interactions identified by the above-described MAGIA^2^ analysis. Specifically, a miRNA was added to a pathway-derived network only if one (or more) of its validated or predicted target genes is a pathway component. Then, a modified recursive version of CliPPER topological pathway analysis [41] was applied to the composite network, as previously described [22] in order to identify the most important and non-redundant circuit modulated across groups. Briefly, (i) in the first step, the most significant pathways were selected using *P* < 0.1 as cut-off value for significance; (ii) for each dataset, the upper-scored 10^th^ percentile of the portion of these previously selected pathways (calculated over a 10,000-permutation step) mostly associated with phenotype were selected; and (iii) for each dataset a meta-pathway was assembled using the pathways extracted from previous step and finally re-analyzed. Finally, the genes/miRNAs included in the upper-scored 10^th^ percentile of the new generated pathways were selected for testing the overlapping between the two datasets.

### Statistical analysis

To compare the distribution of values between two populations, Wilcoxon rank-sum test was applied using standard function in *base* R package. Conventional survival analysis was performed using *survival* package for R software. Cox proportional hazards model in the *globaltest* function in the homonymous package for R software (under 100,000 permutations) was used to test the positive or negative association between miRNA expression levels, assumed as continuous variables, and overall survival (OS) or progression-free survival (PFS) as clinical outcome. Benjamini and Hochberg correction was used for multiple testing adjustments.

## SUPPLEMENTARY MATERIAL FIGURES AND TABLES


