# Supplementary material for: Disentangling the microRNA regulatory milieu in multiple myeloma: integrative genomics analysis outlines mixed miRNA-TF circuits and pathway-derived networks modulated in t(4;14) patients
Source: Oncotarget. 2015 Oct 19;7(3):2367–78. doi: 10.18632/oncotarget.6151 (PMC4823041; doi:10.18632/oncotarget.6151)
Supplement: Supplementary file 1 [file oncotarget-07-2367-s001.pdf]

Disentangling the microRNA regulatory *milieu* in multiple myeloma: integrative genomics analysis outlines mixed miRNA-TF circuits and pathway-derived networks modulated in t(4;14) patients

Supplementary Material

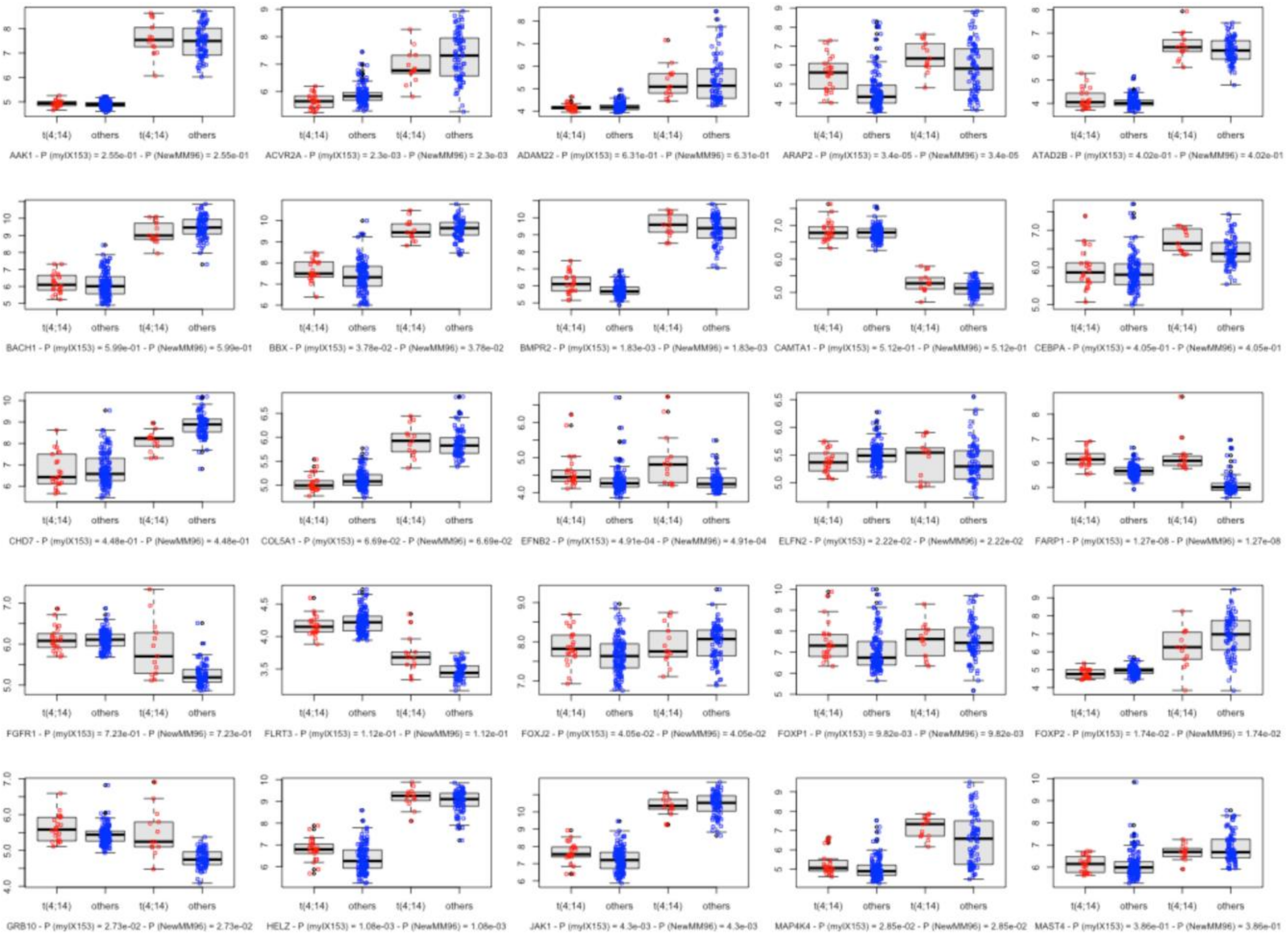

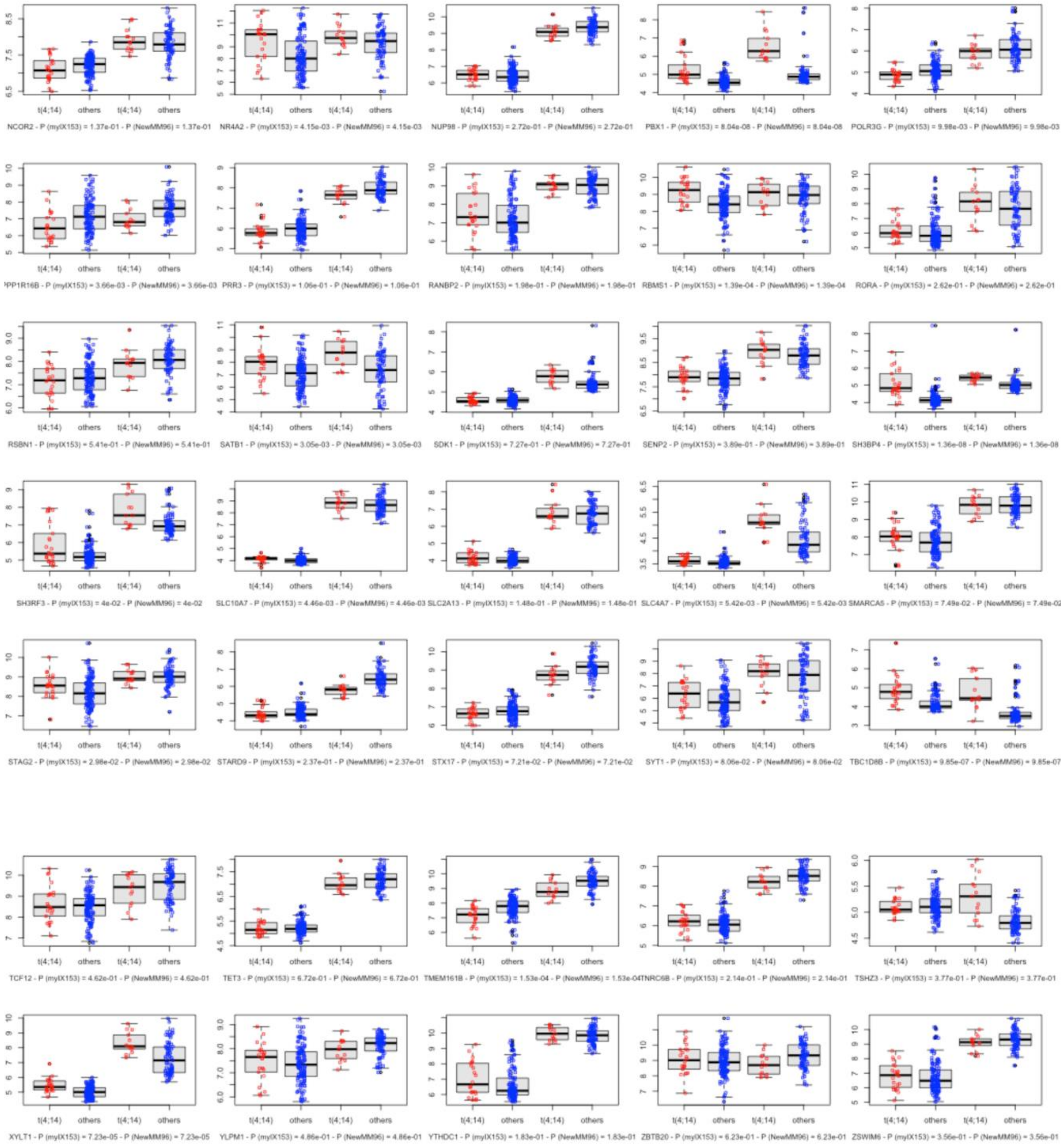

Supplementary Figure 1. Genes and TFs expression level in the network, measured by microarray analysis in the MyIX153 and in the NewMM96 dataset respectively. Red dots in boxplot represent the t(4;14) patient expression, while blue ones represent non-t(4;14) patients expression.

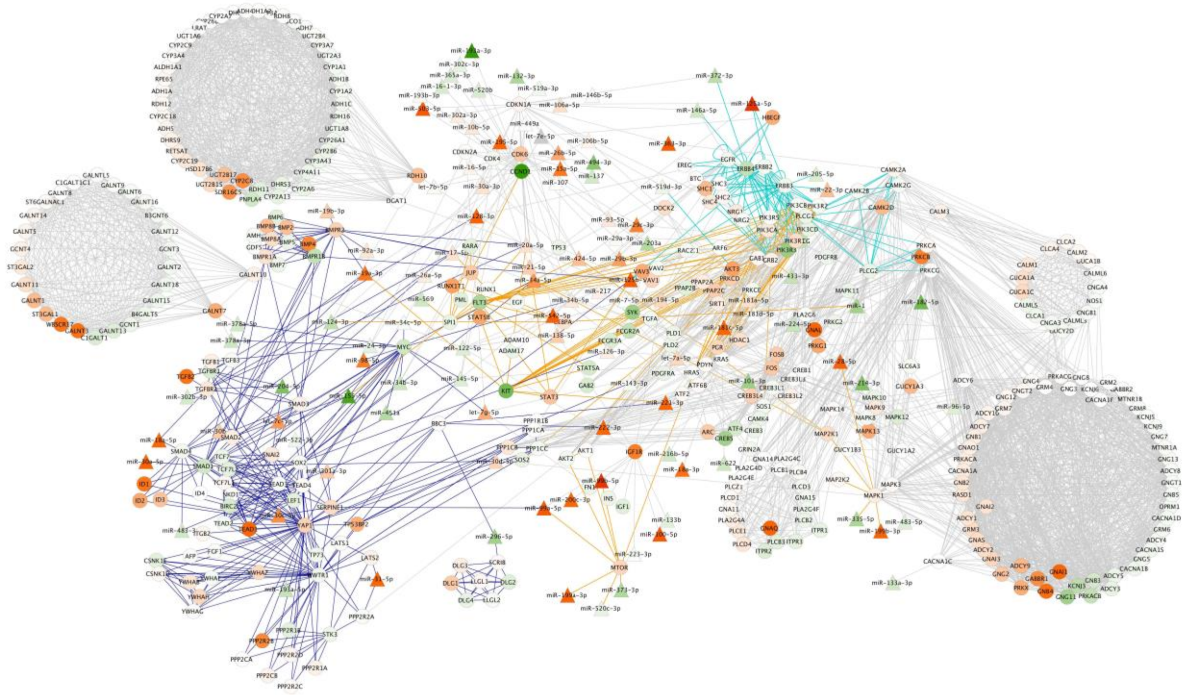

Supplementary Figure 2. Whole union network of the two meta-pathways build on the two and four most significant paths associated with t(4;14) phenotype in MyIX153 and NewMM96 datasets, respectively.

(A)

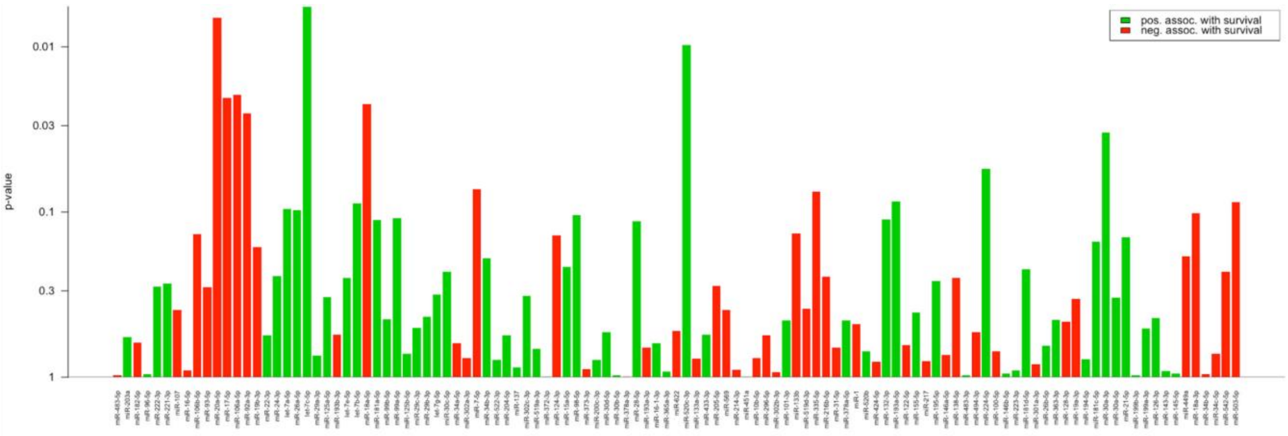

(B)

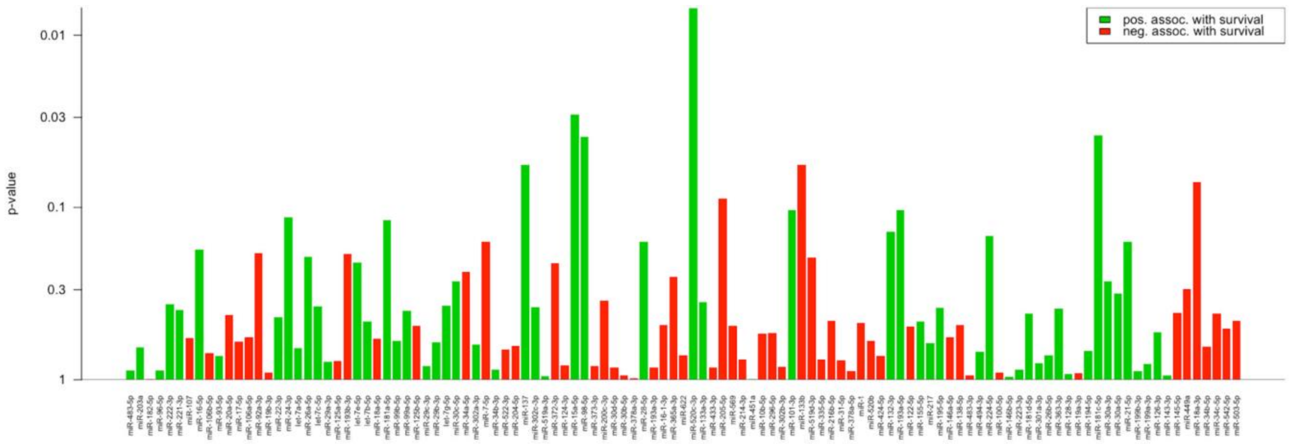

Supplementary Figure 3. (A) Histogram showing the levels of significance of the correlation between each miRNA transcript expression and overall survival in MyIX cases, as obtained after running *globaltest* function in R, using Cox proportional hazards and 100,000 as survival model and number of permutations, respectively. Red bars indicate that higher expression corresponded to poorer outcome. **(B)** Histogram showing the levels of significance of the correlation between each miRNA transcript expression and progression-free survival in MyIX cases, as obtained after running *globaltest* function in R, using Cox proportional hazards and 100,000 as survival model and number of permutations, respectively. Red and green bars indicate that higher or lower expression, respectively, corresponded to poorer outcome.

(A)

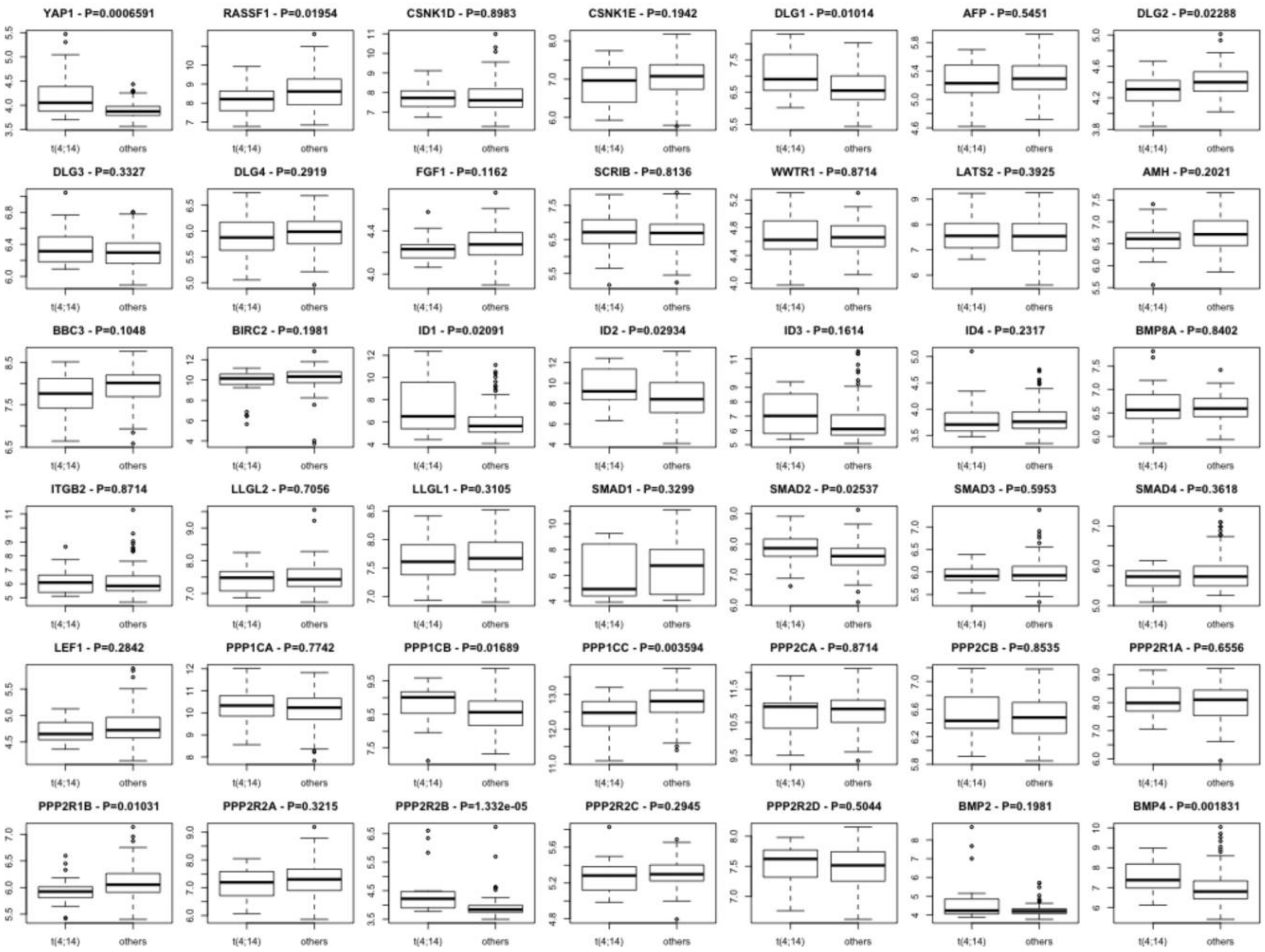
YAP1 - P=0.0006591
RASSF1 - P=0.01954
CSNK1D - P=0.8983
CSNK1E - P=0.1942
DLG1 - P=0.01014
AFP - P=0.5451
DLG2 - P=0.02288
DLG3 - P=0.3327
DLG4 - P=0.2919
FGF1 - P=0.1162
SCRIB - P=0.8136
WWTR1 - P=0.8714
LATS2 - P=0.3925
AMH - P=0.2021
BBC3 - P=0.1048
BIRC2 - P=0.1981
ID1 - P=0.02091
ID2 - P=0.02934
ID3 - P=0.1614
ID4 - P=0.2317
BMP8A - P=0.8402
ITGB2 - P=0.8714
LLGL2 - P=0.7056
LLGL1 - P=0.3105
SMAD1 - P=0.3299
SMAD2 - P=0.02537
SMAD3 - P=0.5953
SMAD4 - P=0.3618
LEF1 - P=0.2842
PPP1CA - P=0.7742
PPP1CB - P=0.01689
PPP1CC - P=0.003594
PPP2CA - P=0.8714
PPP2CB - P=0.8535
PPP2R1A - P=0.6556
PPP2R1B - P=0.01031
PPP2R2A - P=0.3215
PPP2R2B - P=1.332e-05
PPP2R2C - P=0.2945
PPP2R2D - P=0.5044
BMP2 - P=0.1981
BMP4 - P=0.001831
t(4;14)
others

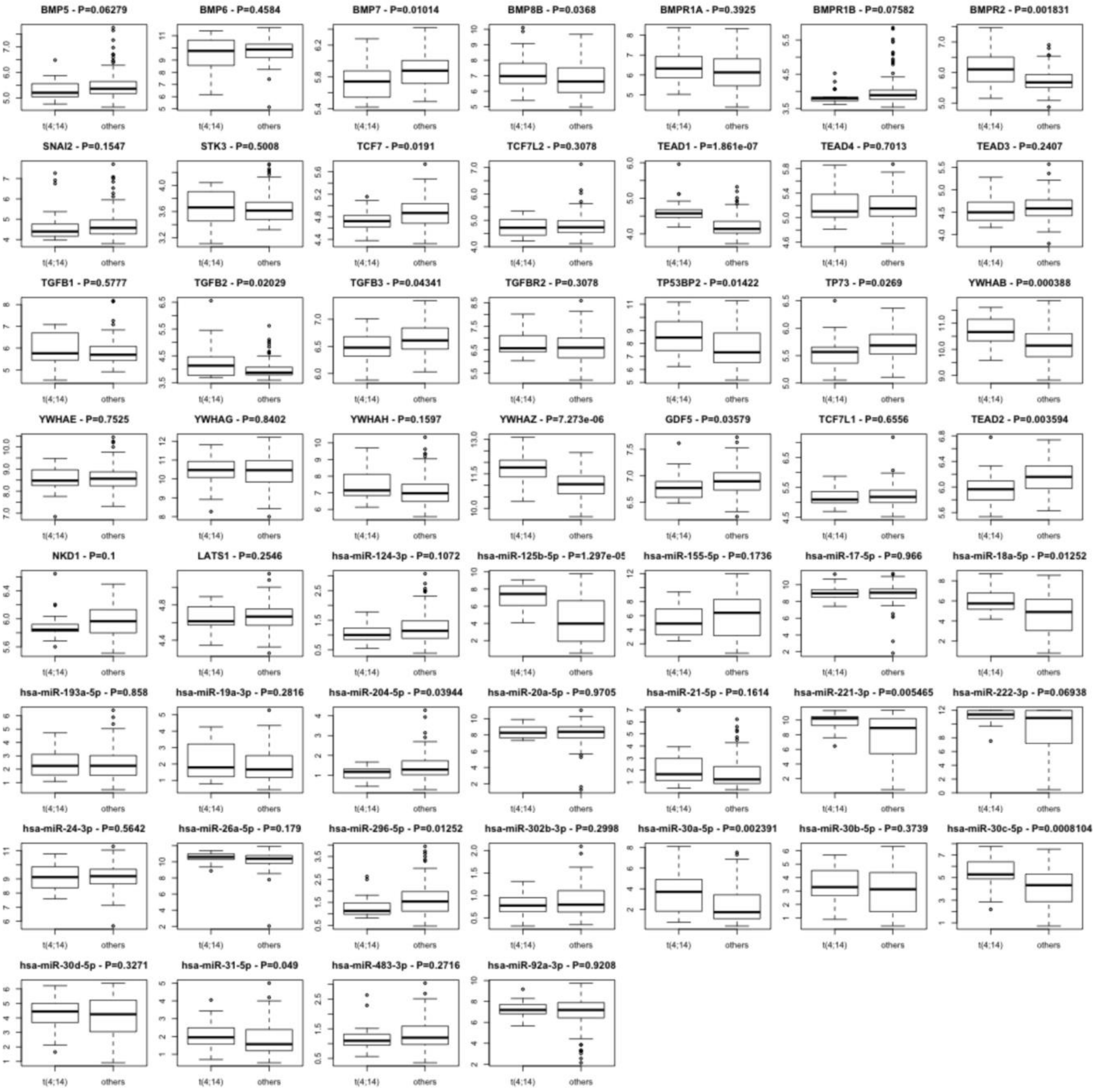

BMP5 - P=0.06279
BMP6 - P=0.4584
BMP7 - P=0.01014
BMP8B - P=0.0368
BMPR1A - P=0.3925
BMPR1B - P=0.07582
BMPR2 - P=0.001831
SNAI2 - P=0.1547
STK3 - P=0.5008
TCF7 - P=0.0191
TCF7L2 - P=0.3078
TEAD1 - P=1.861e-07
TEAD4 - P=0.7013
TEAD3 - P=0.2407
TGFB1 - P=0.5777
TGFB2 - P=0.02029
TGFB3 - P=0.04341
TGFBR2 - P=0.3078
TP53BP2 - P=0.01422
TP73 - P=0.0269
YWHAB - P=0.000388
YWHAE - P=0.7525
YWHAG - P=0.8402
YWHAH - P=0.1597
YWHAZ - P=7.273e-06
GDF5 - P=0.03579
TCF7L1 - P=0.6556
TEAD2 - P=0.003594
NKD1 - P=0.1
LATS1 - P=0.2546
hsa-miR-124-3p - P=0.1072
hsa-miR-125b-5p - P=1.297e-05
hsa-miR-155-5p - P=0.1736
hsa-miR-17-5p - P=0.966
hsa-miR-18a-5p - P=0.01252
hsa-miR-193a-5p - P=0.858
hsa-miR-19a-3p - P=0.2816
hsa-miR-204-5p - P=0.03944
hsa-miR-20a-5p - P=0.9705
hsa-miR-21-5p - P=0.1614
hsa-miR-221-3p - P=0.005465
hsa-miR-222-3p - P=0.06938
hsa-miR-24-3p - P=0.5642
hsa-miR-26a-5p - P=0.179
hsa-miR-296-5p - P=0.01252
hsa-miR-302b-3p - P=0.2998
hsa-miR-30a-5p - P=0.002391
hsa-miR-30b-5p - P=0.3739
hsa-miR-30c-5p - P=0.0008104
hsa-miR-30d-5p - P=0.3271
hsa-miR-31-5p - P=0.049
hsa-miR-483-3p - P=0.2716
hsa-miR-92a-3p - P=0.9208
t(4;14)
others

(B)

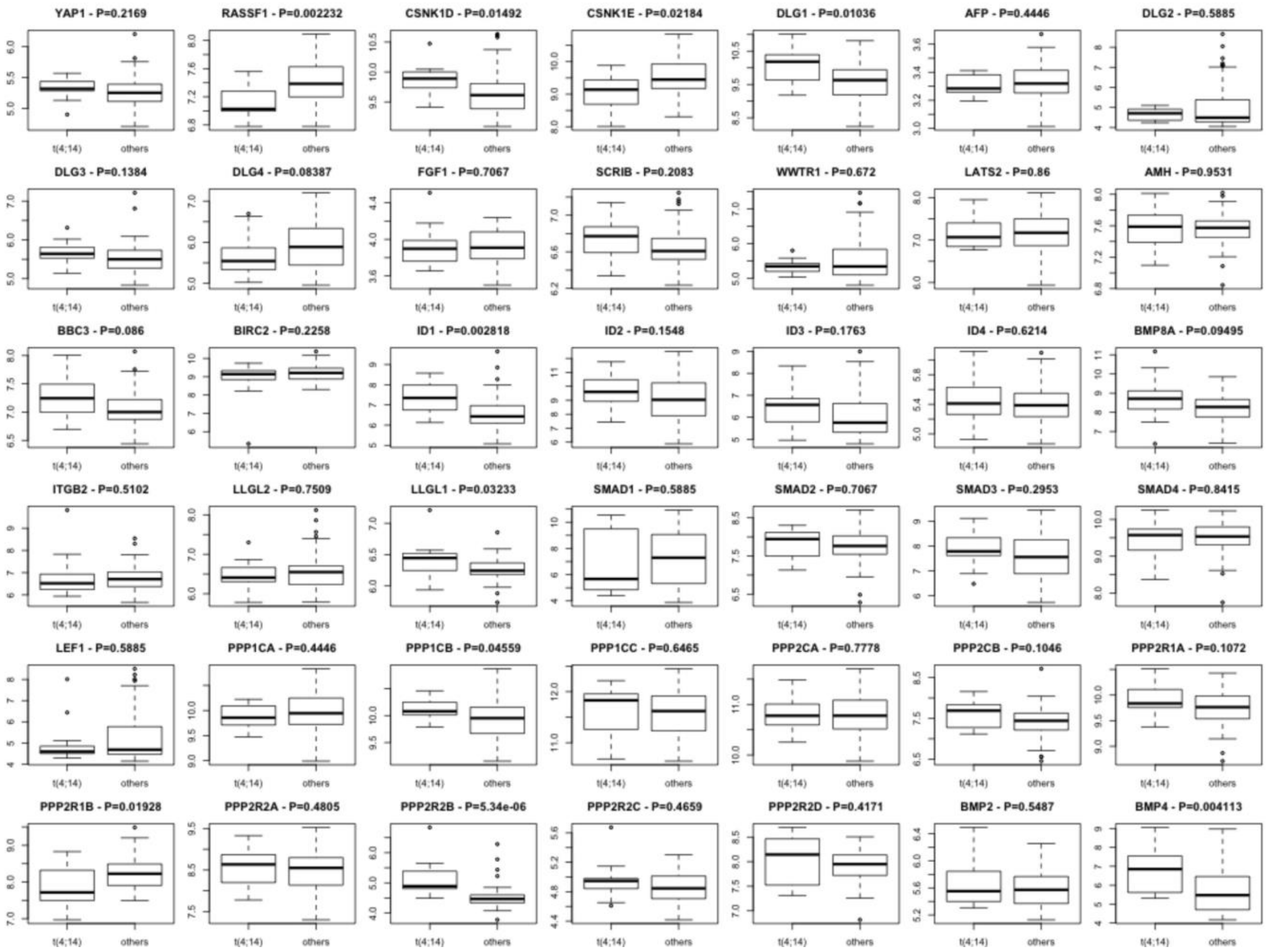

YAP1 - P=0.2169
RASSF1 - P=0.002232
CSNK1D - P=0.01492
CSNK1E - P=0.02184
DLG1 - P=0.01036
AFP - P=0.4446
DLG2 - P=0.5885
DLG3 - P=0.1384
DLG4 - P=0.08387
FGF1 - P=0.7067
SCRIB - P=0.2083
WWTR1 - P=0.672
LATS2 - P=0.86
AMH - P=0.9531
BBC3 - P=0.086
BIRC2 - P=0.2258
ID1 - P=0.002818
ID2 - P=0.1548
ID3 - P=0.1763
ID4 - P=0.6214
BMP8A - P=0.09495
ITGB2 - P=0.5102
LLGL2 - P=0.7509
LLGL1 - P=0.03233
SMAD1 - P=0.5885
SMAD2 - P=0.7067
SMAD3 - P=0.2953
SMAD4 - P=0.8415
LEF1 - P=0.5885
PPP1CA - P=0.4446
PPP1CB - P=0.04559
PPP1CC - P=0.6465
PPP2CA - P=0.7778
PPP2CB - P=0.1046
PPP2R1A - P=0.1072
PPP2R1B - P=0.01928
PPP2R2A - P=0.4805
PPP2R2B - P=5.34e-06
PPP2R2C - P=0.4659
PPP2R2D - P=0.4171
BMP2 - P=0.5487
BMP4 - P=0.004113
t(4;14)
others

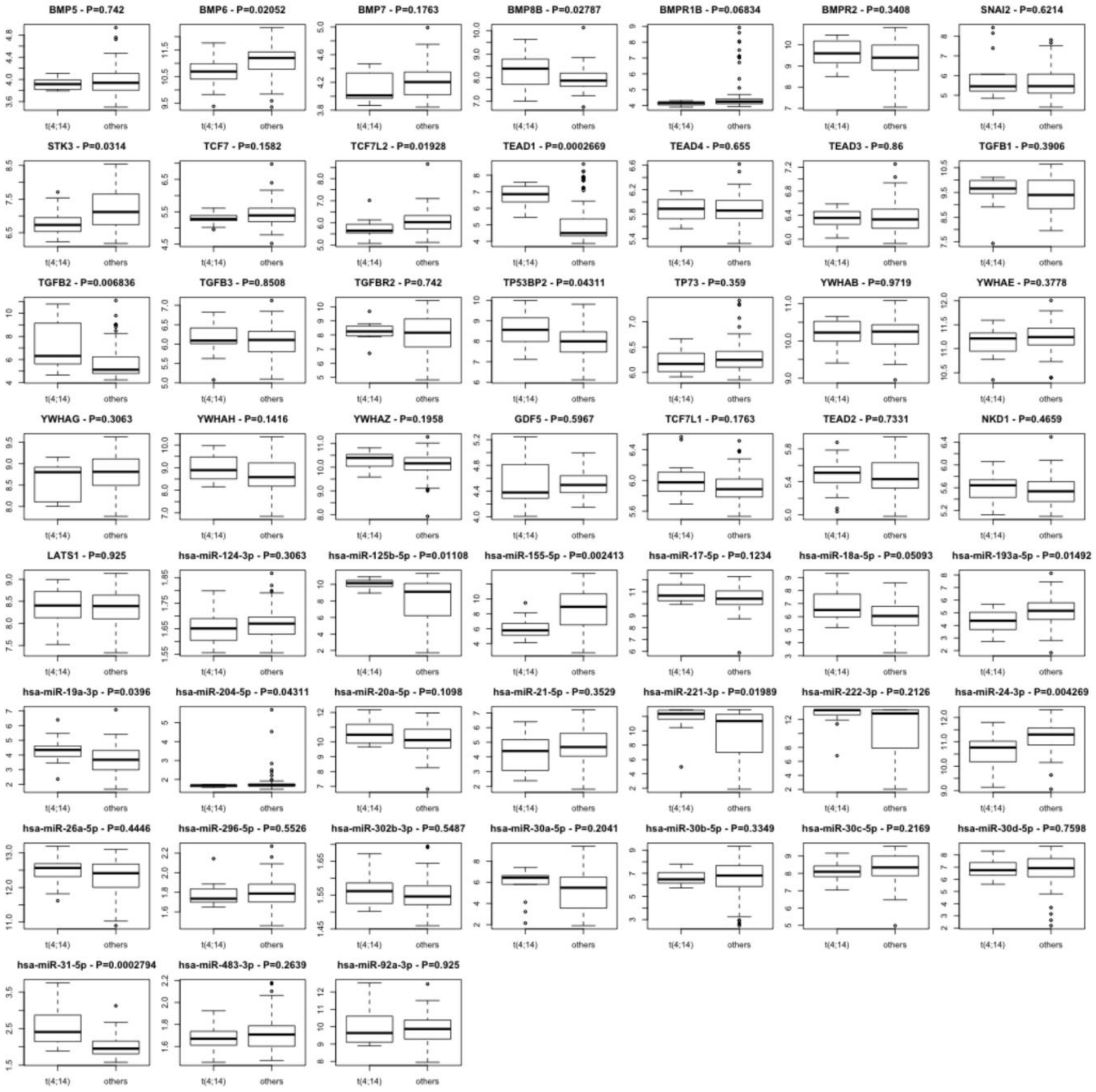

Supplementary Figure 4. (A) Hippo pathway members expression level in MyIX153B. **(B)** Hippo pathway members expression level in NewMM96.

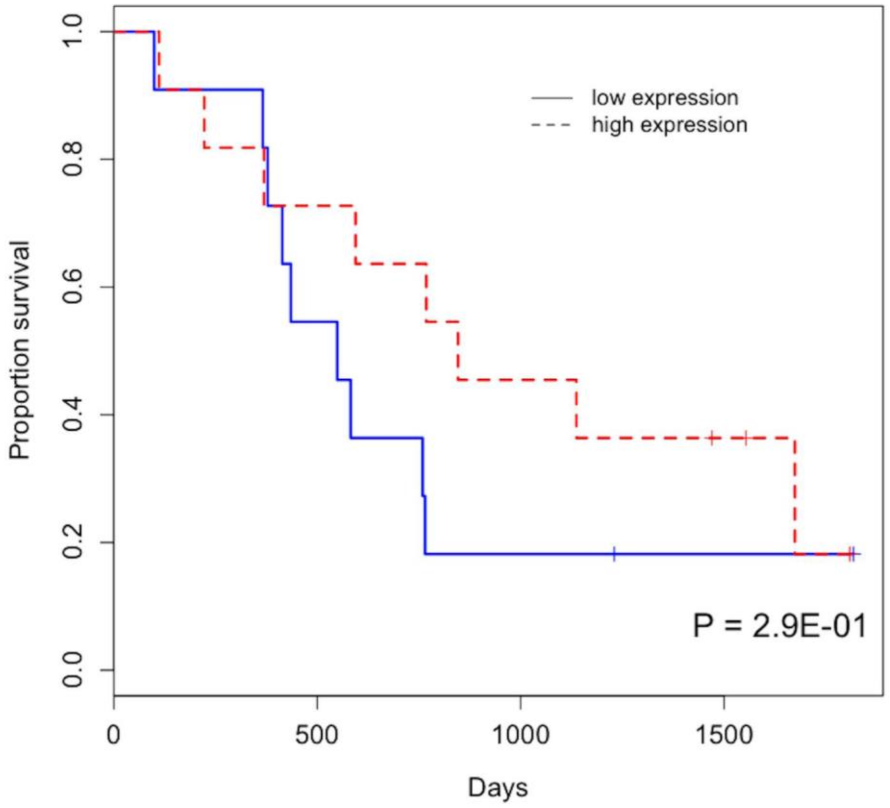

Supplementary Figure 5. Kaplan–Meier curves of the 2 groups defined by *YAP1* expression level (on the median value).

Supplementary Table 1. Significant miRNA/TF/genes triplets identified by MAGIA2 analysis in MyIX153 and NewMM96 datasets

MyIX153											
TF	TF SYMBOL	mRNA	mRNA symbol	miRNA	TF/mRNA measure	TF/mRNA q-value	miRNA/TF measure	miRNA/TF q-value	miRNA /mRNA measure	miRNA/mRNA q-value	
5087	PBX1	64131	XYLT1	hsa-let-7e-5p	0.30901227	0.000637425	0.430122884	9.71E-06	0.235589647	0.174286355	
5087	PBX1	344558	SH3RF3	hsa-let-7e-5p	0.243652712	0.009419121	0.430122884	9.71E-06	0.223523252	0.212352572	
2308	FOXO1	4216	MAP3K4	hsa-mir-15a-5p	0.415212272	1.42E-06	0.259509365	0.054613867	0.320069127	0.017448486	
3659	IRF1	9693	RAPGEF2	hsa-mir-20b-5p	0.596598531	4.20E-14	0.252042585	0.065870305	0.311345682	0.023854884	
7528	YY1	4848	CNOT2	hsa-mir-181c-5p	0.537060171	4.22E-11	0.275686471	0.035670076	0.253715624	0.119729923	
7528	YY1	345778	MTX3	hsa-mir-181c-5p	0.374468907	1.94E-05	0.275686471	0.035670076	0.2763378	0.068589261	
7528	YY1	7182	NR2C2	hsa-mir-181c-5p	0.352393528	6.93E-05	0.275686471	0.035670076	0.201513591	0.305582862	
7528	YY1	157807	CLVS1	hsa-mir-181c-5p	-0.318795579	0.000399167	0.275686471	0.035670076	-0.286952133	0.052501097	
6908	TBP	84458	LCOR	hsa-mir-15a-5p	0.211984649	0.027086129	0.281513231	0.035670076	0.333718728	0.009809755	
2308	FOXO1	403	ARL3	hsa-mir-15a-5p	-0.469931686	2.21E-08	0.259509365	0.054613867	-0.295831697	0.041113627	
3659	IRF1	80055	PGAP1	hsa-mir-20b-5p	0.409151716	2.14E-06	0.252042585	0.065870305	0.293824727	0.044159801	
84159	ARID5B	143684	FAM76B	hsa-mir-32-5p	0.333977683	0.000185428	-0.218521845	0.188969758	-0.289665733	0.04941421	
3659	IRF1	26278	SACS	hsa-mir-20b-5p	0.351610771	7.21E-05	0.252042585	0.065870305	0.286607715	0.052924587	
2308	FOXO1	57700	FAM160B1	hsa-mir-15a-5p	0.544132158	2.00E-11	0.259509365	0.054613867	0.285557917	0.054416531	
2308	FOXO1	54861	SNRK	hsa-mir-15a-5p	0.65878332	5.21E-18	0.259509365	0.054613867	0.28224688	0.058641889	
2308	FOXO1	57532	NUFIP2	hsa-mir-15a-5p	0.63598338	1.78E-16	0.259509365	0.054613867	0.227407919	0.202027857	
2308	FOXO1	27244	SESN1	hsa-mir-15a-5p	0.539417488	3.28E-11	0.259509365	0.054613867	0.244074144	0.148212187	
2308	FOXO1	23405	DICER1	hsa-mir-15a-5p	0.365801269	3.24E-05	0.259509365	0.054613867	0.200806481	0.309998999	
3659	IRF1	51366	UBR5	hsa-mir-20b-5p	0.52903383	9.76E-11	0.252042585	0.065870305	0.255148988	0.116801024	
3659	IRF1	148867	SLC30A7	hsa-mir-20b-5p	-0.462663032	3.99E-08	0.252042585	0.065870305	-0.20287448	0.298459429	
3659	IRF1	23143	LRCH1	hsa-mir-20b-5p	0.410417674	1.96E-06	0.252042585	0.065870305	0.211034746	0.261728768	
7023	TFAP4	4130	MAP1A	hsa-mir-15a-5p	0.448127719	1.26E-07	-0.236022728	0.103931123	-0.230175657	0.191597372	
84159	ARID5B	200576	PIKFYVE	hsa-mir-32-5p	0.378779025	1.49E-05	-0.218521845	0.188969758	-0.246084583	0.143095121	
84159	ARID5B	79668	PARP8	hsa-mir-32-5p	0.374361494	1.95E-05	-0.218521845	0.188969758	-0.223489631	0.212352572	
84159	ARID5B	27031	NPHP3	hsa-mir-32-5p	0.371894825	2.27E-05	-0.218521845	0.188969758	-0.213719449	0.252169905	
1050	CEBPA	10160	FARP1	hsa-let-7e-5p	0.316657887	0.000441661	0.206170301	0.249488848	0.456558771	8.59E-06	
1050	CEBPA	54454	ATAD2B	hsa-let-7e-5p	0.24727436	0.008232341	0.206170301	0.249488848	0.37029516	0.001913337	
2623	GATA1	56134	PCDHAC2	hsa-mir-181c-5p	0.581031621	3.02E-13	-0.312035263	0.020678213	-0.328491943	0.012216187	
55810	FOXJ2	5937	RBMS1	hsa-let-7e-5p	0.241806844	0.010053576	0.220774254	0.193092201	0.360620865	0.003223491	
55810	FOXJ2	571	BACH1	hsa-mir-155-5p	0.384163531	1.08E-05	0.268486456	0.071360458	0.321513898	0.016727268	
55810	FOXJ2	10622	POLR3G	hsa-mir-30a-5p	-0.402148994	3.39E-06	0.221771096	0.193092201	-0.319607587	0.017574286	
2623	GATA1	56145	PCDHA3	hsa-mir-181c-5p	0.621758935	1.40E-15	-0.312035263	0.020678213	-0.254025794	0.118708006	
2623	GATA1	3836	KPNA1	hsa-mir-181c-5p	-0.544861895	1.85E-11	-0.312035263	0.020678213	0.211309883	0.261096382	
2623	GATA1	9857	CEP350	hsa-mir-181c-5p	-0.505082347	1.02E-09	-0.312035263	0.020678213	0.248882462	0.134588203	
2623	GATA1	56143	PCDHA5	hsa-mir-181c-5p	0.504493234	1.08E-09	-0.312035263	0.020678213	-0.253456583	0.120371517	
2623	GATA1	51441	YTHDF2	hsa-mir-181c-5p	-0.480524438	9.18E-09	-0.312035263	0.020678213	0.245098465	0.145751715	
2623	GATA1	11052	CPSF6	hsa-mir-181c-5p	-0.469385387	2.30E-08	-0.312035263	0.020678213	0.216519379	0.242639182	
2623	GATA1	55023	PHIP	hsa-mir-181c-5p	-0.466094097	3.01E-08	-0.312035263	0.020678213	0.215249909	0.24619449	
2623	GATA1	285527	FRYL	hsa-mir-181c-5p	-0.445501453	1.54E-07	-0.312035263	0.020678213	0.2060026	0.285510466	
2623	GATA1	9774	BCLAF1	hsa-mir-181c-5p	-0.445463465	1.55E-07	-0.312035263	0.020678213	0.202826473	0.298459429	
2623	GATA1	26047	CNTNAP2	hsa-mir-181c-5p	0.42788589	5.75E-07	-0.312035263	0.020678213	-0.214849956	0.247505212	
2623	GATA1	868	CBLB	hsa-mir-181c-5p	-0.416988451	1.26E-06	-0.312035263	0.020678213	0.251659569	0.126200087	
2623	GATA1	987	LRBA	hsa-mir-181c-5p	-0.378734192	1.50E-05	-0.312035263	0.020678213	0.217653226	0.239091439	
2623	GATA1	6249	CLIP1	hsa-mir-181c-5p	-0.37624225	1.74E-05	-0.312035263	0.020678213	0.244437306	0.148002899	
2623	GATA1	254827	NAALADL2	hsa-mir-181c-5p	0.370668574	2.44E-05	-0.312035263	0.020678213	-0.282774159	0.058451195	
2623	GATA1	1362	CPD	hsa-mir-181c-5p	-0.369192683	2.66E-05	-0.312035263	0.020678213	0.221720823	0.220020913	
2623	GATA1	56135	PCDHAC1	hsa-mir-181c-5p	0.359107855	4.78E-05	-0.312035263	0.020678213	-0.283795975	0.05642727	
2623	GATA1	23608	MKRN1	hsa-mir-181c-5p	-0.256488736	0.00586148	-0.312035263	0.020678213	0.204376941	0.292960481	
55810	FOXJ2	92	ACVR2A	hsa-mir-155-5p	0.283141248	0.002021207	0.268486456	0.071360458	0.309097541	0.025805429	
55810	FOXJ2	6304	SATB1	hsa-mir-30a-5p	0.224434429	0.018232493	0.221771096	0.193092201	0.324352755	0.014574582	
6927	HNF1A	3207	HOXA11	hsa-mir-181c-5p	0.625890912	7.64E-16	-0.285655948	0.040387133	-0.248419257	0.136473595	
6927	HNF1A	84188	FAR1	hsa-mir-181c-5p	-0.48106389	8.77E-09	-0.285655948	0.040387133	0.215736854	0.245095731	
6927	HNF1A	26047	CNTNAP2	hsa-mir-181c-5p	0.429095496	5.29E-07	-0.285655948	0.040387133	-0.214849956	0.247505212	
6927	HNF1A	285527	FRYL	hsa-mir-181c-5p	-0.41920026	1.08E-06	-0.285655948	0.040387133	0.2060026	0.285510466	
6927	HNF1A	84458	LCOR	hsa-mir-181c-5p	-0.382703365	1.18E-05	-0.285655948	0.040387133	0.211900004	0.25965577	
6927	HNF1A	987	LRBA	hsa-mir-181c-5p	-0.373085909	2.11E-05	-0.285655948	0.040387133	0.217653226	0.239091439	
6927	HNF1A	5604	MAP2K1	hsa-mir-181c-5p	-0.332552111	0.00019972	-0.285655948	0.040387133	0.233338649	0.180317061	
6927	HNF1A	91526	ANKRD44	hsa-mir-181c-5p	-0.309369939	0.000627979	-0.285655948	0.040387133	0.223292933	0.213536572	
6927	HNF1A	284273	ZADH2	hsa-mir-181c-5p	-0.301224768	0.000911997	-0.285655948	0.040387133	0.268674869	0.085040448	
4609	MYC	10018	BCL2L11	hsa-mir-20b-5p	0.374670375	1.92E-05	0.223931669	0.183203498	0.290463737	0.047912775	
4609	MYC	26278	SACS	hsa-mir-20b-5p	0.339473315	0.000139012	0.223931669	0.183203498	0.286607715	0.052924587	
55810	FOXJ2	55636	CHD7	hsa-mir-155-5p	0.479290075	1.01E-08	0.268486456	0.071360458	0.244808691	0.14638679	
55810	FOXJ2	57688	ZSWIM6	hsa-mir-155-5p	0.326074779	0.00027725	0.268486456	0.071360458	0.216024498	0.244055633	
55810	FOXJ2	6095	RORA	hsa-mir-155-5p	0.311330843	0.000573756	0.268486456	0.071360458	0.246685859	0.141328154	
5966	REL	7170	TPM3	hsa-mir-18a-5p	0.565769773	1.82E-12	0.216357247	0.205694111	0.273385708	0.073741143	
55810	FOXJ2	54665	RSBN1	hsa-mir-216a-5p	0.369757039	2.57E-05	-0.220708171	0.193092201	-0.270045686	0.081023787	
55810	FOXJ2	9931	HELZ	hsa-let-7e-5p	0.424831092	7.17E-07	0.220774254	0.193092201	0.266237259	0.088333706	
55810	FOXJ2	9448	MAP4K4	hsa-let-7e-5p	0.370792213	2.42E-05	0.220774254	0.193092201	0.261918418	0.099044977	
4609	MYC	1844	DUSP2	hsa-mir-20b-5p	0.33895008	0.000142504	0.223931669	0.183203498	0.259558597	0.103303987	
55810	FOXJ2	23112	TNRC6B	hsa-mir-30a-5p	0.325174236	0.000290096	0.221771096	0.193092201	0.254765322	0.117350074	
55810	FOXJ2	8467	SMARCA5	hsa-mir-99a-5p	0.397729485	4.54E-06	0.240270446	0.124081481	0.227883715	0.20014152	
4609	MYC	27327	TNRC6A	hsa-mir-20b-5p	0.362129039	4.01E-05	0.223931669	0.183203498	0.243857429	0.148431426	
55810	FOXJ2	116984	ARAP2	hsa-mir-30a-5p	0.424095137	7.57E-07	0.221771096	0.193092201	0.23395889	0.178359287	
55810	FOXJ2	5903	RANBP2	hsa-let-7e-5p	0.401178382	3.61E-06	0.220774254	0.193092201	0.232680376	0.182598592	
5966	REL	6789	STK4	hsa-mir-18a-5p	0.371585087	2.31E-05	0.216357247	0.205694111	0.231834488	0.184938589	
55810	FOXJ2	3716	JAK1	hsa-mir-30a-5p	0.568964518	1.25E-12	0.221771096	0.193092201	0.212262856	0.258053429	
55810	FOXJ2	91746	YTHDC1	hsa-mir-30a-5p	0.503046962	1.22E-09	0.221771096	0.193092201	0.203252906	0.297316409	
55810	FOXJ2	56252	YLPM1	hsa-mir-216a-5p	0.497888813	1.97E-09	-0.220708171	0.193092201	-0.215002613	0.247315432	
55810	FOXJ2	10735	STAG2	hsa-mir-30a-5p	0.471641921	1.92E-08	0.221771096	0.193092201	0.205069189	0.289744168	
55810	FOXJ2	27086	FOXP1	hsa-let-7e-5p	0.415000284	1.44E-06	0.220774254	0.193092201	0.229733857	0.193133624	
55810	FOXJ2	56987	BBX	hsa-mir-30a-5p	0.391356326	6.87E-06	0.221771096	0.193092201	0.215443887	0.245830146	

NewMM96										
TF	TF SYMBOL	mRNA	mRNA symbol	miRNA	TF/mRNA measure	TF/mRNA q-value	miRNA/TF measure	miRNA/TF q-value	miRNA /mRNA measure	miRNA/mRNA q-value
1869	E2F1	2908	NR3C1	hsa-mir-18a-5p	-0.338675024	0.009284758	0.277637145	0.239694427	-0.436733525	0.009366627
1869	E2F1	2908	NR3C1	hsa-mir-18b-5p	-0.338675024	0.009284758	0.277637145	0.239694427	-0.436733525	0.009366627
6927	HNF1A	64327	LMBR1	hsa-mir-30d-5p	-0.45272825	0.000188189	-0.220948968	0.386809501	0.382605226	0.038002898
1050	CEBPA	54885	TBC1D8B	hsa-mir-125a-5p	0.274360853	0.044648484	0.254471429	0.301126003	0.462874423	0.003780892
6722	SRF	6095	RORA	hsa-mir-193b-3p	0.331100611	0.011365517	-0.27206523	0.245812218	-0.366175878	0.054298279
3131	HLF	6095	RORA	hsa-mir-30d-5p	0.361892981	0.004773709	-0.234001599	0.360066545	-0.357692976	0.063654211
1050	CEBPA	4928	NUP98	hsa-let-7e-5p	-0.486641177	4.35E-05	0.346582897	0.068597635	-0.263297374	0.257757063

4609	MYC	5976	UPF1	hsa-mir-221-3p	0.384436368	0.002330279	0.246904038	0.326683913	0.351590306	0.071881911
55810	FOXJ2	200424	TET3	hsa-mir-133b	0.597903312	8.13E-08	0.243342981	0.339228518	0.350076589	0.074643502
1050	CEBPA	93986	FOXP2	hsa-let-7e-5p	-0.344026364	0.008015639	0.346582897	0.068597635	-0.212378274	0.415868472
1050	CEBPA	153396	TMEM161B	hsa-mir-125a-5p	-0.639900247	3.70E-09	0.254471429	0.301126003	-0.342894967	0.082760535
1050	CEBPA	2260	FGFR1	hsa-mir-125a-5p	0.243337203	0.082449854	0.254471429	0.301126003	0.458176138	0.004261106
3131	HLF	360023	ZBTB41	hsa-mir-30d-5p	-0.333619259	0.010649736	-0.234001599	0.360066545	0.345946804	0.078440895
4609	MYC	22895	RPH3A	hsa-mir-221-3p	0.29838223	0.025822686	0.246904038	0.326683913	0.357445916	0.063699446
6927	HNF1A	9690	UBE3C	hsa-mir-30d-5p	-0.237464786	0.091883768	-0.220948968	0.386809501	0.437463578	0.009188772
6908	TBP	9448	MAP4K4	hsa-mir-30a-5p	0.247530942	0.075856063	0.227388751	0.37288596	0.399670925	0.025734693
6908	TBP	9444	QKI	hsa-mir-30a-5p	0.533337574	4.16E-06	0.227388751	0.37288596	0.330513399	0.103382066
6908	TBP	57507	ZNF608	hsa-mir-30a-5p	0.304693714	0.022132859	0.227388751	0.37288596	0.342828032	0.082819862
1050	CEBPA	2887	GRB10	hsa-mir-125a-5p	0.22518908	0.114516674	0.254471429	0.301126003	0.57904415	1.89E-05
55810	FOXJ2	6857	SYT1	hsa-mir-133b	0.367030221	0.004084063	0.243342981	0.339228518	-0.323396357	0.116651098
6722	SRF	9874	TLK1	hsa-mir-214-3p	0.592001715	1.21E-07	0.319574777	0.122022239	0.292132877	0.179646182
6722	SRF	9697	TRAM2	hsa-mir-214-3p	0.521998134	7.82E-06	0.319574777	0.122022239	0.295312029	0.172215344
6722	SRF	26137	ZBTB20	hsa-mir-214-3p	0.457505073	0.000153391	0.319574777	0.122022239	0.22429058	0.37634305
5966	REL	57493	HEG1	hsa-mir-27a-3p	0.39065528	0.001886997	-0.219458387	0.392171841	-0.31988294	0.122493242
6927	HNF1A	4810	NHS	hsa-mir-30d-5p	0.536414749	3.50E-06	-0.220948968	0.386809501	-0.314713983	0.132346236
55810	FOXJ2	659	BMPR2	hsa-mir-99a-5p	0.628743487	8.88E-09	0.288900715	0.220506396	0.311229918	0.139245529
6908	TBP	5335	PLCG1	hsa-mir-30a-5p	0.512976752	1.23E-05	0.227388751	0.37288596	0.305117092	0.151105091
5966	REL	80223	RAB11FIP1	hsa-mir-27a-3p	0.283940776	0.036139933	-0.219458387	0.392171841	-0.320799582	0.120374572
6722	SRF	257194	NEGR1	hsa-mir-150-5p	0.360828272	0.004903453	-0.27465886	0.243673328	-0.304172542	0.152176324
6908	TBP	8658	TNKS	hsa-mir-30a-5p	0.242289735	0.084191339	0.227388751	0.37288596	0.350576795	0.073763164
6722	SRF	23327	NEDD4L	hsa-mir-193b-3p	0.36339463	0.004585052	-0.27206523	0.245812218	-0.297610302	0.165810301
3131	HLF	11113	CIT	hsa-mir-30d-5p	-0.239210162	0.088941621	-0.234001599	0.360066545	0.341988281	0.084296952
1050	CEBPA	221935	SDK1	hsa-let-7e-5p	0.20548181	0.158914195	0.346582897	0.068597635	0.329792404	0.104662541
4609	MYC	80205	CHD9	hsa-mir-22-3p	-0.333275216	0.010756488	-0.263648296	0.275511935	0.296627558	0.168109278
4609	MYC	10527	IPO7	hsa-mir-22-3p	0.430572797	0.000456313	-0.263648296	0.275511935	-0.290714056	0.183617684
4779	NFE2L1	6547	SLC8A3	hsa-mir-21-5p	-0.202627123	0.165796564	0.203434083	0.456849188	-0.401406897	0.024547948
1958	EGR1	80205	CHD9	hsa-mir-92b-3p	0.219116562	0.12724628	-0.266469266	0.262866647	-0.354611656	0.067283672
55810	FOXJ2	84068	SLC10A7	hsa-mir-204-5p	0.585227087	1.88E-07	-0.263236318	0.275511935	-0.285520093	0.194674023
5966	REL	2113	ETS1	hsa-mir-193a-3p	0.246099229	0.078042957	0.304778617	0.164315296	0.322622906	0.1175815
6927	HNF1A	219623	TMEM26	hsa-mir-30d-5p	0.574809702	3.65E-07	-0.220948968	0.386809501	-0.28491861	0.196643764
6886	TAL1	10735	STAG2	hsa-mir-363-3p	-0.230310655	0.104548054	-0.326061697	0.101500172	0.211594118	0.418471902
55810	FOXJ2	27086	FOXP1	hsa-mir-449b-5p	0.427582024	0.000512301	-0.257551747	0.293235229	-0.278182934	0.214524324
5966	REL	6925	TCF4	hsa-mir-193a-3p	-0.229425975	0.106228171	0.304778617	0.164315296	-0.326000646	0.111368891
55810	FOXJ2	4929	NR4A2	hsa-mir-449b-5p	0.3154115	0.017059098	-0.257551747	0.293235229	-0.282914711	0.201153906
55810	FOXJ2	9612	NCOR2	hsa-mir-449b-5p	0.64249249	2.97E-09	-0.257551747	0.293235229	-0.276615151	0.218261219
6927	HNF1A	51804	SIX4	hsa-mir-30d-5p	0.342893859	0.008259446	-0.220948968	0.386809501	-0.275806015	0.221149748
55810	FOXJ2	114134	SLC2A13	hsa-mir-449b-5p	0.408196229	0.001039602	-0.257551747	0.293235229	-0.270109284	0.237192474
55810	FOXJ2	375449	MAST4	hsa-mir-214-3p	0.372412765	0.003457634	0.214427938	0.405220009	0.270628211	0.235380109
6722	SRF	57534	MIB1	hsa-mir-150-5p	-0.493648744	3.14E-05	-0.27465886	0.243673328	0.264457269	0.253631178
6777	STAT5B	50650	ARHGEF3	hsa-mir-17-5p	0.356709566	0.005537673	0.20840242	0.4329936	-0.268444357	0.241799575
6722	SRF	51773	RSF1	hsa-mir-193b-3p	-0.383764889	0.002379255	-0.27206523	0.245812218	-0.248667113	0.299754271
6722	SRF	10409	BASP1	hsa-mir-150-5p	0.343180403	0.00819254	-0.27465886	0.243673328	-0.206487276	0.436388366
6722	SRF	7003	TEAD1	hsa-mir-150-5p	0.341054488	0.008707235	-0.27465886	0.243673328	-0.220632654	0.38730255
5966	REL	57493	HEG1	hsa-mir-17-5p	0.39065528	0.001886997	0.269862665	0.250836654	0.210536015	0.422391715
6722	SRF	5578	PRKCA	hsa-mir-150-5p	0.300764227	0.024416383	-0.27465886	0.243673328	-0.203515305	0.446484364
6927	HNF1A	57724	EPG5	hsa-mir-30d-5p	-0.208880795	0.150888914	-0.220948968	0.386809501	0.319747537	0.122691415
55810	FOXJ2	22848	AAK1	hsa-mir-204-5p	0.474778989	7.38E-05	-0.263236318	0.275511935	-0.216173564	0.402627662
1958	EGR1	659	BMPR2	hsa-mir-92b-3p	0.324495137	0.013590067	-0.266469266	0.262866647	-0.223140193	0.380609759
55810	FOXJ2	59343	SENP2	hsa-mir-214-3p	-0.58833297	1.53E-07	0.214427938	0.405220009	-0.253610515	0.284924838
55810	FOXJ2	200424	TET3	hsa-mir-214-3p	0.597903312	8.13E-08	0.214427938	0.405220009	0.253402125	0.285209987
3131	HLF	2104	ESRRG	hsa-mir-30d-5p	0.207609613	0.153739459	-0.234001599	0.360066545	-0.313511746	0.134009507
55810	FOXJ2	6938	TCF12	hsa-mir-449b-5p	0.632619514	6.58E-09	-0.257551747	0.293235229	-0.219982469	0.389229344
5966	REL	57493	HEG1	hsa-mir-19a-3p	0.39065528	0.001886997	0.223461304	0.381194072	0.251274877	0.291865123
55810	FOXJ2	53616	ADAM22	hsa-mir-449b-5p	-0.338085269	0.009436203	-0.257551747	0.293235229	0.242600076	0.318275935
1050	CEBPA	23677	SH3BP4	hsa-mir-125a-5p	0.319492301	0.015407101	0.254471429	0.301126003	0.22472741	0.374432085
6908	TBP	23314	SATB2	hsa-mir-30a-5p	0.332990356	0.010831044	0.227388751	0.37288596	0.229852714	0.358594197
55810	FOXJ2	26137	ZBTB20	hsa-mir-214-3p	0.430520762	0.000456406	0.214427938	0.405220009	0.22429058	0.37634305
6927	HNF1A	81706	PPP1R14C	hsa-mir-30d-5p	0.573214496	4.03E-07	-0.220948968	0.386809501	-0.221784879	0.384125725
6908	TBP	114882	OSBPL8	hsa-mir-30a-5p	0.308107276	0.020418287	0.227388751	0.37288596	0.226458075	0.369009666
5966	REL	3021	H3F3B	hsa-mir-27a-3p	0.526320952	6.19E-06	-0.219458387	0.392171841	-0.20085688	0.455273337
1050	CEBPA	1289	COL5A1	hsa-mir-192-5p	0.712297721	4.07E-12	-0.208035838	0.4329936	-0.204320716	0.443033137
55810	FOXJ2	23261	CAMTA1	hsa-mir-100-5p	0.305775301	0.021564352	0.203032549	0.458205671	0.21095776	0.42066609
3659	IRF1	11113	CIT	hsa-mir-130b-3p	-0.515852184	1.07E-05	-0.413125718	0.006737956	0.289251809	0.185760362
3659	IRF1	22990	PCNX	hsa-mir-130b-3p	0.491925146	3.40E-05	-0.413125718	0.006737956	-0.280239669	0.208230843
5087	PBX1	344558	SH3RF3	hsa-let-7e-5p	0.478935546	6.19E-05	0.419509568	0.006737956	0.261890293	0.262356366
5087	PBX1	64131	XYLT1	hsa-let-7e-5p	0.42189404	0.000633711	0.419509568	0.006737956	0.261378096	0.263271123
3659	IRF1	3782	KCNN3	hsa-mir-130b-3p	-0.402974758	0.001248697	-0.413125718	0.006737956	0.298572635	0.163163284
5087	PBX1	57519	STARD9	hsa-let-7e-5p	-0.28481417	0.035415882	0.419509568	0.006737956	-0.478740315	0.002231699
3659	IRF1	9575	CLOCK	hsa-mir-130b-3p	-0.351040729	0.006565523	-0.413125718	0.006737956	0.251010804	0.291865123
3659	IRF1	11177	BAZ1A	hsa-mir-130b-3p	0.32463226	0.013546266	-0.413125718	0.006737956	-0.209905337	0.424179688
3659	IRF1	57614	KIAA1468	hsa-mir-130b-3p	-0.313399338	0.017895432	-0.413125718	0.006737956	0.230601634	0.356068764
5087	PBX1	221935	SDK1	hsa-let-7e-5p	0.308693518	0.020121177	0.419509568	0.006737956	0.329792404	0.104662541
6722	SRF	2060	EPS15	hsa-mir-181b-5p	-0.299107977	0.025400917	0.219208487	0.573477739	-0.464472788	0.003615759
3659	IRF1	94134	ARHGAP12	hsa-mir-130b-3p	-0.297620141	0.026264295	-0.413125718	0.006737956	0.346803589	0.077498769
3659	IRF1	4302	MLLT6	hsa-mir-130b-3p	0.293102579	0.029239627	-0.413125718	0.006737956	-0.319583734	0.122691415
3659	IRF1	9839	ZEB2	hsa-mir-130b-3p	0.291825603	0.030193834	-0.413125718	0.006737956	-0.209597436	0.424694747
3659	IRF1	6916	TBXAS1	hsa-mir-130b-3p	0.288732838	0.032531974	-0.413125718	0.006737956	-0.275598133	0.221868048
3659	IRF1	9917	FAM20B	hsa-mir-130b-3p	-0.284911277	0.03535492	-0.413125718	0.006737956	0.268888286	0.240940924
3131	HLF	116931	MED12L	hsa-mir-148b-3p	0.388137032	0.002057149	-0.295017472	0.18692269	-0.372256635	0.048613643
3659	IRF1	79932	KIAA0319L	hsa-mir-130b-3p	-0.273677824	0.045406184	-0.413125718	0.006737956	0.248621642	0.300024593
3659	IRF1	7111	TMOD1	hsa-mir-130b-3p	0.266924698	0.052032323	-0.413125718	0.006737956	-0.301602082	0.155414216
5087	PBX1	9497	SLC4A7	hsa-let-7e-5p	0.266707266	0.052190565	0.419509568	0.006737956	0.264327627	0.254353424
4779	NFE2L1	2104	ESRRG	hsa-mir-195-5p	-0.262347037	0.057235342	0.398954193	0.008380966	-0.294467895	0.174333663
3131	HLF	6095	RORA	hsa-mir-30d-5p	0.361892981	0.004773709	-0.234001599	0.500789619	-0.357692976	0.063654211
1050	CEBPA	23767	FLRT3	hsa-mir-101-3p	0.393402401	0.001742893	-0.295848959	0.18692269	-0.35321638	0.0699262
5087	PBX1	26051	PPP1R16B	hsa-let-7e-5p	-0.221997649	0.120988395	0.419509568	0.006737956	-0.356568528	0.065121172
4779	NFE2L1	2104	ESRRG	hsa-mir-497-5p	-0.262347037	0.057235342	0.372037361	0.019071879	-0.325823119	0.111639886
3659	IRF1	22849	CPEB3	hsa-mir-130b-3p	-0.242410486	0.084018089	-0.413125718	0.006737956	0.346059811	0.078270756
3131	HLF	360023	ZBTB41	hsa-mir-30d-5p	-0.333619259	0.010649736	-0.234001599	0.500789619	0.345946804	0.078440895
3659	IRF1	22834	ZNF652	hsa-mir-130b-3p	-0.229696326	0.105736991	-0.413125718	0.006737956	0.253696415	0.28462153

3659	IRF1	6059	ABCE1	hsa-mir-130b-3p	-0.225454813	0.114130536	-0.413125718	0.006737956	0.263382328	0.257669989
6722	SRF	7049	TGFBR3	hsa-mir-181b-5p	-0.235839738	0.094579058	0.219208487	0.573477739	-0.393027226	0.031022509
7026	NR2F2	5594	MAPK1	hsa-mir-194-5p	-0.498089222	2.55E-05	-0.21897886	0.573477739	0.315068692	0.131794602
3659	IRF1	4774	NFIA	hsa-mir-130b-3p	0.218384993	0.128877847	-0.413125718	0.006737956	-0.258888181	0.27046487
3659	IRF1	80854	SETD7	hsa-mir-130b-3p	-0.217873096	0.129848524	-0.413125718	0.006737956	0.282811704	0.201288037
3131	HLF	6095	RORA	hsa-mir-148b-3p	0.361892981	0.004773709	-0.295017472	0.18692269	-0.314808905	0.132159875
5087	PBX1	93986	FOXP2	hsa-let-7e-5p	-0.215574455	0.135031042	0.419509568	0.006737956	-0.212378274	0.415868472
3659	IRF1	11069	RAPGEF4	hsa-mir-130b-3p	0.212036284	0.143185494	-0.413125718	0.006737956	-0.225983426	0.370344769
5087	PBX1	55014	STX17	hsa-let-7e-5p	-0.210545827	0.146781658	0.419509568	0.006737956	-0.229803709	0.358607459
3659	IRF1	3479	IGF1	hsa-mir-130b-3p	0.207819001	0.153277523	-0.413125718	0.006737956	-0.20767668	0.431997867
3131	HLF	11113	CIT	hsa-mir-30d-5p	-0.239210162	0.088941621	-0.234001599	0.500789619	0.341988281	0.084296952
6722	SRF	9908	G3BP2	hsa-mir-181b-5p	-0.555189861	1.25E-06	0.219208487	0.573477739	-0.293377357	0.177354732
6722	SRF	22884	WDR37	hsa-mir-181b-5p	-0.313454221	0.017878734	0.219208487	0.573477739	-0.298896034	0.162757307
1050	CEBPA	80742	PRR3	hsa-mir-101-3p	-0.522862324	7.47E-06	-0.295848959	0.18692269	0.212758327	0.414430956
1050	CEBPA	1948	EFNB2	hsa-mir-101-3p	0.454829693	0.00017182	-0.295848959	0.18692269	-0.241713914	0.321417831
1050	CEBPA	57616	TSHZ3	hsa-mir-101-3p	0.433069226	0.000415438	-0.295848959	0.18692269	-0.278630581	0.213564378
1050	CEBPA	114794	ELFN2	hsa-mir-101-3p	0.332759357	0.010880308	-0.295848959	0.18692269	-0.217964645	0.396116772
3659	IRF1	57678	GPAM	hsa-mir-183-5p	-0.58964358	1.40E-07	-0.212218405	0.573861965	0.28326411	0.19978114
6722	SRF	26750	RPS6KC1	hsa-mir-22-3p	-0.458920005	0.000143977	-0.280711927	0.214848181	0.278960938	0.212326959
6722	SRF	149420	PDIK1L	hsa-mir-22-3p	-0.62923334	8.82E-09	-0.280711927	0.214848181	0.221108958	0.385763051
6722	SRF	26127	FGFR1OP2	hsa-mir-22-3p	-0.507383441	1.63E-05	-0.280711927	0.214848181	0.201658263	0.452405133
55810	FOXJ2	27086	FOXP1	hsa-mir-449b-5p	0.427582024	0.000512301	-0.257551747	0.364777946	-0.278182934	0.214524324
6722	SRF	9880	ZBTB39	hsa-mir-22-3p	0.449363628	0.000214724	-0.280711927	0.214848181	-0.244857249	0.312082992
6722	SRF	152485	ZNF827	hsa-mir-22-3p	0.443996939	0.000270316	-0.280711927	0.214848181	-0.249618878	0.295971114
55810	FOXJ2	4929	NR4A2	hsa-mir-449b-5p	0.3154115	0.017059098	-0.257551747	0.364777946	-0.282914711	0.201153906
55810	FOXJ2	9612	NCOR2	hsa-mir-449b-5p	0.64249249	2.97E-09	-0.257551747	0.364777946	-0.276615151	0.218261219
6722	SRF	26052	DNM3	hsa-mir-22-3p	0.36231774	0.004720651	-0.280711927	0.214848181	-0.201600669	0.452405133
55810	FOXJ2	114134	SLC2A13	hsa-mir-449b-5p	0.408196229	0.001039602	-0.257551747	0.364777946	-0.270109284	0.237192474
1958	EGR1	57620	STIM2	hsa-mir-181b-5p	-0.200220302	0.171913962	0.224799986	0.573477739	-0.345329886	0.079315208
3131	HLF	2104	ESRRG	hsa-mir-30d-5p	0.207609613	0.153739459	-0.234001599	0.500789619	-0.313511746	0.134009507
3131	HLF	2104	ESRRG	hsa-mir-148b-3p	0.207609613	0.153739459	-0.295017472	0.18692269	-0.303985551	0.152264627
55810	FOXJ2	53616	ADAM22	hsa-mir-449b-5p	-0.338085269	0.009436203	-0.257551747	0.364777946	0.242600076	0.318275935
55810	FOXJ2	6938	TCF12	hsa-mir-449b-5p	0.632619514	6.58E-09	-0.257551747	0.364777946	-0.219982469	0.389229344
3659	IRF1	8502	PKP4	hsa-mir-183-5p	-0.356884672	0.005510432	-0.212218405	0.573861965	0.226729527	0.368183936
6722	SRF	23253	ANKRD12	hsa-mir-181b-5p	0.371378762	0.003560796	0.219208487	0.573477739	-0.211198349	0.419930268
6722	SRF	4299	AFF1	hsa-mir-181b-5p	0.498323	2.53E-05	0.219208487	0.573477739	-0.206586093	0.435887141
6722	SRF	56147	PCDHA1	hsa-mir-181b-5p	0.318148449	0.015923734	0.219208487	0.573477739	0.208327391	0.429969251
1958	EGR1	5813	PURA	hsa-mir-506-3p	0.545608549	2.16E-06	-0.206234775	0.573861965	-0.20363594	0.446186755
6722	SRF	257194	NEGR1	hsa-mir-181b-5p	0.360828272	0.004903453	0.219208487	0.573477739	0.200984129	0.454697296

Supplementary Table 2. (A) 66 and **(B)** 47 most significantly modulated pathways identified at *P*<0.1, according to Micrographite analysis of NewMM96 and MyIX153 dataset, respectively. The 17 common pathways are underlined.

A.

	p-value mean	*q*-value mean	*p*-value variance	*q*-value variance
**Acute myeloid leukemia**	< 0.01	< 0.01	< 0.01	< 0.01
**Amoebiasis**	< 0.01	< 0.01	< 0.01	< 0.01
**Amphetamine addiction**	< 0.01	< 0.01	< 0.01	< 0.01
**Apoptosis**	< 0.01	< 0.01	< 0.01	< 0.01
**Basal cell carcinoma**	< 0.01	< 0.01	< 0.01	< 0.01
**Bladder cancer**	< 0.01	< 0.01	< 0.01	< 0.01
**Calcium signaling pathway**	< 0.01	< 0.01	< 0.01	< 0.01
**Cholinergic synapse**	< 0.01	< 0.01	< 0.01	< 0.01
**Circadian entrainment**	< 0.01	< 0.01	< 0.01	< 0.01
**Cytosolic DNA-sensing pathway**	0.01	0.013898305	< 0.01	< 0.01
**Dilated cardiomyopathy**	< 0.01	< 0.01	< 0.01	< 0.01
**Dopaminergic synapse**	< 0.01	< 0.01	< 0.01	< 0.01
**Dorso-ventral axis formation**	< 0.01	< 0.01	< 0.01	< 0.01
**Endometrial cancer**	< 0.01	< 0.01	< 0.01	< 0.01
**Epstein-Barr virus infection**	< 0.01	< 0.01	< 0.01	< 0.01
**ErbB signaling pathway**	< 0.01	< 0.01	< 0.01	< 0.01
**Fc gamma R-mediated phagocytosis**	< 0.01	< 0.01	< 0.01	< 0.01
**Fructose and mannose metabolism**	< 0.01	< 0.01	< 0.01	< 0.01
**Gap junction**	< 0.01	< 0.01	< 0.01	< 0.01
**Glioma**	< 0.01	< 0.01	< 0.01	< 0.01
**Long-term depression**	< 0.01	< 0.01	< 0.01	< 0.01
**Morphine addiction**	< 0.01	< 0.01	< 0.01	< 0.01
**Pancreatic secretion**	< 0.01	< 0.01	< 0.01	< 0.01
**Primary bile acid biosynthesis**	< 0.01	< 0.01	< 0.01	< 0.01
**Progesterone-mediated oocyte maturation**	< 0.01	< 0.01	< 0.01	< 0.01
**Rheumatoid arthritis**	< 0.01	< 0.01	< 0.01	< 0.01
**Selenocompound metabolism**	0.01	0.013898305	< 0.01	< 0.01
**Thyroid cancer**	< 0.01	< 0.01	< 0.01	< 0.01
**Transcriptional misregulation in cancer**	< 0.01	< 0.01	< 0.01	< 0.01
**Type II diabetes mellitus**	< 0.01	< 0.01	< 0.01	< 0.01
**Viral myocarditis**	< 0.01	< 0.01	< 0.01	< 0.01
**Bacterial invasion of epithelial cells**	< 0.01	< 0.01	0.01	0.041
**Colorectal cancer**	< 0.01	< 0.01	0.01	0.041
**Fatty acid biosynthesis**	0.02	0.026666667	0.01	0.041
**Glutamatergic synapse**	< 0.01	< 0.01	0.01	0.041
**Long-term potentiation**	< 0.01	< 0.01	0.01	0.041
**Sphingolipid metabolism**	< 0.01	< 0.01	0.01	0.041
**Vasopressin-regulated water reabsorption**	< 0.01	< 0.01	0.01	0.041
**Vibrio cholerae infection**	< 0.01	< 0.01	0.01	0.041
**Amino sugar and nucleotide sugar metabolism**	< 0.01	< 0.01	0.02	0.0656
**B cell receptor signaling pathway**	< 0.01	< 0.01	0.02	0.0656

Chagas disease (American trypanosomiasis)	< 0.01	< 0.01	0.02	0.0656
ECM-receptor interaction	< 0.01	< 0.01	0.02	0.0656
Epithelial cell signaling in Helicobacter pylori infection	< 0.01	< 0.01	0.02	0.0656
Inositol phosphate metabolism	< 0.01	< 0.01	0.02	0.0656
Mucin type O-Glycan biosynthesis	< 0.01	< 0.01	0.02	0.0656
NOD-like receptor signaling pathway	< 0.01	< 0.01	0.02	0.0656
Phosphatidylinositol signaling system	< 0.01	< 0.01	0.02	0.0656
Retrograde endocannabinoid signaling	< 0.01	< 0.01	0.02	0.0656
Adherens junction	< 0.01	< 0.01	0.03	0.083389831
African trypanosomiasis	< 0.01	< 0.01	0.03	0.083389831
Alcoholism	< 0.01	< 0.01	0.03	0.083389831
Glycolysis / Gluconeogenesis	< 0.01	< 0.01	0.03	0.083389831
Glycosphingolipid biosynthesis - ganglio series	0.01	0.013898305	0.03	0.083389831
Intestinal immune network for IgA production	< 0.01	< 0.01	0.03	0.083389831
Mineral absorption	0.05	0.061654135	0.03	0.083389831
Pentose phosphate pathway	< 0.01	< 0.01	0.03	0.083389831
beta-Alanine metabolism	0.01	0.013898305	0.04	0.096470588
Complement and coagulation cascades	< 0.01	< 0.01	0.04	0.096470588
Fc epsilon RI signaling pathway	< 0.01	< 0.01	0.04	0.096470588
GnRH signaling pathway	< 0.01	< 0.01	0.04	0.096470588
Legionellosis	0.03	0.038740157	0.04	0.096470588
Pathogenic Escherichia coli infection	< 0.01	< 0.01	0.04	0.096470588
RIG-I-like receptor signaling pathway	< 0.01	< 0.01	0.04	0.096470588
Salmonella infection	< 0.01	< 0.01	0.04	0.096470588
Serotonergic synapse	< 0.01	< 0.01	0.04	0.096470588

B.

	p-value Mean	*q*-value Mean	*p*-value variance	*q*-value variance
Adipocytokine signaling pathway	< 0.01	< 0.01	< 0.01	< 0.01
Alcoholism	< 0.01	< 0.01	< 0.01	< 0.01
Amphetamine addiction	< 0.01	< 0.01	< 0.01	< 0.01
Chagas disease (American trypanosomiasis)	< 0.01	< 0.01	< 0.01	< 0.01
Chemical carcinogenesis	< 0.01	< 0.01	< 0.01	< 0.01
Cocaine addiction	< 0.01	< 0.01	< 0.01	< 0.01
Drug metabolism - cytochrome P450	< 0.01	< 0.01	< 0.01	< 0.01
Epithelial cell signaling in Helicobacter pylori infection	< 0.01	< 0.01	< 0.01	< 0.01
Fat digestion and absorption	< 0.01	< 0.01	< 0.01	< 0.01
Fatty acid biosynthesis	< 0.01	< 0.01	< 0.01	< 0.01
GABAergic synapse	< 0.01	< 0.01	< 0.01	< 0.01
Glutamatergic synapse	< 0.01	< 0.01	< 0.01	< 0.01
Glycine, serine and threonine metabolism	< 0.01	< 0.01	< 0.01	< 0.01
Hippo signaling pathway	< 0.01	< 0.01	< 0.01	< 0.01
Lysine degradation	< 0.01	< 0.01	< 0.01	< 0.01
Metabolism of xenobiotics by cytochrome P450	< 0.01	< 0.01	< 0.01	< 0.01
Mineral absorption	< 0.01	< 0.01	< 0.01	< 0.01

Morphine addiction	< 0.01	< 0.01	< 0.01	< 0.01
Mucin type O-Glycan biosynthesis	< 0.01	< 0.01	< 0.01	< 0.01
Phenylalanine metabolism	< 0.01	< 0.01	< 0.01	< 0.01
Phototransduction	< 0.01	< 0.01	< 0.01	< 0.01
Progesterone-mediated oocyte maturation	< 0.01	< 0.01	< 0.01	< 0.01
Regulation of actin cytoskeleton	< 0.01	< 0.01	< 0.01	< 0.01
Retinol metabolism	< 0.01	< 0.01	< 0.01	< 0.01
Vasopressin-regulated water reabsorption	< 0.01	< 0.01	< 0.01	< 0.01
Alzheimer's disease	< 0.01	< 0.01	0.01	0.04775
Arrhythmogenic right ventricular cardiomyopathy (ARVC)	< 0.01	< 0.01	0.01	0.04775
Basal cell carcinoma	< 0.01	< 0.01	0.01	0.04775
Cell adhesion molecules (CAMs)	< 0.01	< 0.01	0.01	0.04775
Circadian entrainment	< 0.01	< 0.01	0.01	0.04775
Citrate cycle (TCA cycle)	< 0.01	< 0.01	0.01	0.04775
Hedgehog signaling pathway	< 0.01	< 0.01	0.01	0.04775
Influenza A	< 0.01	< 0.01	0.01	0.04775
Leishmaniasis	< 0.01	< 0.01	0.01	0.04775
Pathogenic Escherichia coli infection	< 0.01	< 0.01	0.01	0.04775
Sphingolipid metabolism	< 0.01	< 0.01	0.01	0.04775
Steroid biosynthesis	< 0.01	< 0.01	0.01	0.04775
Steroid hormone biosynthesis	< 0.01	< 0.01	0.01	0.04775
Type II diabetes mellitus	< 0.01	< 0.01	0.01	0.04775
Viral myocarditis	< 0.01	< 0.01	0.01	0.04775
Carbohydrate digestion and absorption	< 0.01	< 0.01	0.02	0.081276596
Endocrine and other factor-regulated calcium reabsorption	< 0.01	< 0.01	0.02	0.081276596
Glutathione metabolism	< 0.01	< 0.01	0.02	0.081276596
Leukocyte transendothelial migration	< 0.01	< 0.01	0.02	0.081276596
Notch signaling pathway	< 0.01	< 0.01	0.02	0.081276596
Olfactory transduction	< 0.01	< 0.01	0.02	0.081276596
Phenylalanine, tyrosine and tryptophan biosynthesis	0.03	0.033705882	0.02	0.081276596

Supplementary Table 3A. Globaltest analysis of miRNA expression level and overall survival in MyIX153 dataset. Metrics of correlation using Cox proportional hazards model and 100,000 permutations are reported for each miRNAs.

	p-value	q-value	Statistic	Expected	Std.dev
hsa-let-7c-5p	0.0057	0.36536667	47.5713015	4.55041897	9.89415368
hsa-miR-20a-5p	0.00665	0.36536667	35.6077866	4.53727014	7.36636418
hsa-miR-520c-3p	0.0097	0.36536667	25.0404323	4.52760084	5.37053781
hsa-miR-106a-5p	0.01958	0.40647714	27.7883663	4.54954639	7.50977835
hsa-miR-17-5p	0.02038	0.40647714	25.7373892	4.5544273	6.9308241
hsa-miR-18a-5p	0.0222	0.40647714	28.4685007	4.57399511	8.02625205
hsa-miR-92a-3p	0.02518	0.40647714	31.9947969	4.5630765	9.70657386
hsa-miR-30a-3p	0.03291	0.46485375	12.315713	4.55355513	3.35505459
hsa-miR-224-5p	0.05496	0.58462091	16.8563321	4.58848924	6.22628125
hsa-miR-7-5p	0.07249	0.58462091	16.7291997	4.54913331	7.32903097
hsa-miR-335-5p	0.07545	0.58462091	15.060276	4.54444398	6.53926899
hsa-miR-193a-5p	0.08636	0.58462091	14.6696811	4.56632283	6.83419406
hsa-miR-503-5p	0.08726	0.58462091	16.7038982	4.54531353	8.3331544
hsa-let-7b-5p	0.08848	0.58462091	15.533172	4.54549237	7.57381728
hsa-let-7a-5p	0.0957	0.58462091	14.3538075	4.55855635	7.35536672
hsa-miR-26a-5p	0.0977	0.58462091	18.5922267	4.51879514	10.5175172
hsa-miR-18a-3p	0.10168	0.58462091	15.3836299	4.56213229	8.38729353
hsa-miR-98-5p	0.10384	0.58462091	12.2792582	4.53788305	6.12446572
hsa-miR-99a-5p	0.10879	0.58462091	15.2816927	4.5997991	8.83198124
hsa-miR-132-3p	0.1107	0.58462091	13.449635	4.51539837	7.60391871
hsa-miR-181a-5p	0.11196	0.58462091	25.9884944	4.57281343	17.5113876
hsa-miR-28-5p	0.11382	0.58462091	13.8535935	4.58508678	7.94854278
hsa-miR-133b	0.13519	0.61945731	9.75732631	4.54971189	5.39288392
hsa-miR-106b-5p	0.1358	0.61945731	9.91891297	4.57565985	5.58271074
hsa-miR-124-3p	0.13874	0.61945731	10.1601068	4.56655554	5.91776277
hsa-miR-21-5p	0.14253	0.61945731	8.53399046	4.5682351	4.14650781
hsa-miR-181c-5p	0.15093	0.63167	10.8940981	4.57050253	7.56421674
hsa-miR-19b-3p	0.16331	0.6590725	9.11081228	4.53493779	6.13709047
hsa-miR-449a	0.18553	0.71920733	9.68380171	4.54938163	7.49272831
hsa-miR-34b-3p	0.19094	0.71920733	7.26628992	4.53036577	4.75507712
hsa-miR-15a-5p	0.21436	0.74588921	8.16444999	4.54961508	7.58121517
hsa-miR-181d-5p	0.22222	0.74588921	7.14976765	4.55400926	5.62741978
hsa-miR-542-5p	0.23061	0.74588921	6.88016156	4.53571382	6.36252929
hsa-miR-30c-5p	0.23069	0.74588921	6.99531553	4.53683174	6.49218606
hsa-miR-24-3p	0.24374	0.74588921	6.80347309	4.50746439	7.38031364
hsa-miR-216b-5p	0.24704	0.74588921	7.3595293	4.54706686	8.25003189
hsa-miR-138-5p	0.24944	0.74588921	6.28516354	4.5263898	5.73407317
hsa-let-7e-5p	0.25083	0.74588921	6.70091323	4.53289869	7.69677718
hsa-miR-195-5p	0.26134	0.74616791	6.47207885	4.56824271	7.97870146
hsa-miR-221-3p	0.27101	0.74616791	7.14485583	4.53582445	10.9660231
hsa-miR-205-5p	0.27937	0.74616791	5.78104418	4.54496163	6.59418216
hsa-miR-222-3p	0.28272	0.74616791	8.02852243	4.5060229	13.7653347
hsa-miR-93-5p	0.28394	0.74616791	5.6103906	4.5654188	6.69733256
hsa-let-7g-5p	0.31655	0.78866938	5.50059586	4.57614383	10.8360553
hsa-miR-302c-3p	0.32002	0.78866938	4.80241935	4.52856256	3.82517183
hsa-miR-125a-5p	0.32819	0.78866938	4.67214692	4.52536653	6.6148648
hsa-miR-30a-5p	0.33076	0.78866938	4.60568049	4.58039184	4.86264441
hsa-miR-19a-3p	0.33501	0.78866938	4.46234519	4.54116184	6.06274998
hsa-miR-519d-3p	0.38407	0.86770706	3.83078688	4.5252202	5.22321069
hsa-miR-107	0.39052	0.86770706	3.29622505	4.56169471	8.2041177
hsa-miR-569	0.39162	0.86770706	3.13005377	4.55134598	9.7260149
hsa-miR-155-5p	0.4046	0.87922692	3.18648078	4.53082808	6.94800126
hsa-miR-29b-3p	0.43136	0.88254915	2.63951934	4.5427497	8.02456624
hsa-miR-126-3p	0.4389	0.88254915	2.61734454	4.5588561	7.22423248
hsa-miR-99b-5p	0.44447	0.88254915	1.80106781	4.5059117	10.3731123
hsa-miR-363-3p	0.44856	0.88254915	3.07905901	4.52269354	5.12908493
hsa-miR-378a-5p	0.4516	0.88254915	2.70377024	4.55964133	6.38222064
hsa-miR-101-3p	0.45432	0.88254915	2.83362546	4.55693456	5.94036778
hsa-miR-128-3p	0.4608	0.88254915	2.81078988	4.5448772	5.61616379
hsa-miR-1	0.47593	0.89633483	1.87723504	4.51791477	8.2981564
hsa-miR-29c-3p	0.50402	0.90167543	1.97981053	4.54874931	6.74913511
hsa-miR-199a-3p	0.50689	0.90167543	2.65577849	4.55676509	5.02103164
hsa-miR-622	0.52465	0.90167543	1.27051189	4.54808017	8.14374493

hsa-miR-30d-5p	0.53119	0.90167543	2.15579962	4.54977683	5.88671822
hsa-miR-494-3p	0.53299	0.90167543	1.55982247	4.56050135	8.54723886
hsa-miR-193b-3p	0.55208	0.90167543	2.09767633	4.561033	5.78392166
hsa-miR-433-3p	0.55295	0.90167543	2.06195137	4.53982133	5.42046537
hsa-miR-204-5p	0.55475	0.90167543	1.11895831	4.5252165	7.61601748
hsa-miR-22-3p	0.55645	0.90167543	2.03044564	4.512762	5.7478866
hsa-miR-296-5p	0.55856	0.90167543	2.14266569	4.52641991	5.3723928
hsa-miR-203a	0.57235	0.91092324	2.42219462	4.54845768	5.10030014
hsa-miR-182-5p	0.61466	0.94771878	1.6342414	4.54956799	5.6275083
hsa-miR-16-1-3p	0.62013	0.94771878	1.13784728	4.52800126	6.52440522
hsa-miR-34a-5p	0.62063	0.94771878	1.12441028	4.54149855	6.75302837
hsa-miR-122-5p	0.64004	0.95945692	1.12065478	4.49802521	6.16714015
hsa-miR-26b-5p	0.64697	0.95945692	1.83264731	4.52597017	4.85959794
hsa-miR-193a-3p	0.66206	0.95945692	0.96707281	4.56564222	6.53770908
hsa-miR-31-5p	0.66228	0.95945692	0.89131175	4.53681643	6.52455805
hsa-miR-519a-3p	0.67284	0.96241671	2.04716067	4.54282118	4.46980181
hsa-miR-100-5p	0.69658	0.97505049	1.34765512	4.59191078	5.28043067
hsa-miR-520b	0.69893	0.97505049	0.01794487	4.5460946	7.59406338
hsa-miR-34c-5p	0.72116	0.97708989	0.22301336	4.51736006	6.90885672
hsa-miR-125b-5p	0.72286	0.97708989	0.72816218	4.58018513	6.1245045
hsa-miR-146a-5p	0.73257	0.97708989	0.16001909	4.53017585	6.96374562
hsa-miR-29a-3p	0.73674	0.97708989	0.26302708	4.55997736	6.82293704
hsa-miR-302a-3p	0.76289	0.97708989	0.03802766	4.5005218	6.85084447
hsa-miR-10b-5p	0.76562	0.97708989	1.44264193	4.54229557	4.67664078
hsa-miR-133a-3p	0.77378	0.97708989	0.20381296	4.52402938	6.33796046
hsa-miR-194-5p	0.77847	0.97708989	0.70299681	4.53544359	5.68148196
hsa-miR-522-3p	0.7856	0.97708989	0.88271009	4.58144701	5.50032559
hsa-miR-200c-3p	0.78686	0.97708989	0.28771942	4.55497352	6.33018293
hsa-miR-217	0.79846	0.97856785	0.43062391	4.52887583	5.89663567
hsa-miR-424-5p	0.80537	0.97856785	0.00402515	4.55133382	6.58952342
hsa-miR-301a-3p	0.83523	0.99411	0.03500053	4.52206539	5.93599465
hsa-miR-137	0.86955	0.99411	0.72999714	4.55599461	4.91571099
hsa-miR-373-3p	0.88934	0.99411	0.75875046	4.52146796	4.94203618
hsa-miR-214-3p	0.89913	0.99411	0.5012432	4.54458789	4.88575869
hsa-miR-223-3p	0.90984	0.99411	0.17253701	4.53709948	5.51576337
hsa-miR-16-5p	0.91134	0.99411	0.56541137	4.55648704	5.08623632
hsa-miR-143-3p	0.91674	0.99411	0.35015833	4.54417963	5.22528641
hsa-miR-365a-3p	0.92277	0.99411	0.00097258	4.51151469	5.62246047
hsa-miR-302b-3p	0.93165	0.99411	0.03080135	4.5523542	5.43425849
hsa-miR-145-5p	0.94743	0.99411	9.23E-05	4.53969241	5.06315817
hsa-miR-146b-5p	0.95173	0.99411	0.41085933	4.55894843	4.86781672
hsa-miR-34b-5p	0.95391	0.99411	0.12660025	4.53013378	4.60696766
hsa-miR-96-5p	0.95707	0.99411	0.55567166	4.5283834	4.75181627
hsa-miR-483-3p	0.97125	0.99411	0.06226096	4.53063412	4.38749387
hsa-miR-199b-3p	0.97347	0.99411	0.24056527	4.55609891	4.92461685
hsa-miR-30b-5p	0.97508	0.99411	0.00095375	4.53886615	5.11628673
hsa-miR-483-5p	0.97571	0.99411	0.00843942	4.53382722	4.80322909
hsa-miR-372-3p	0.99057	0.99411	0.33907682	4.54543314	4.30437195
hsa-miR-378a-3p	0.99217	0.99411	0.20864554	4.53584425	4.27127633
hsa-miR-451a	0.99411	0.99411	0.0003744	4.54415951	4.34821564

Supplementary Table 3B. Globaltest analysis of miRNA expression level and progression-free survival in MyIX153 dataset. Metrics of correlation using Cox proportional hazards model and 100,000 permutations are reported for each miRNAs.

	p-value	q-value	Statistic	Expected	Std.dev
hsa-miR-520c-3p	0.00694	0.78422	30.8045102	4.51368816	6.36590262
hsa-miR-15a-5p	0.02886	0.98505182	25.7388974	4.49772608	8.0247851
hsa-miR-181c-5p	0.03825	0.98505182	24.3849675	4.52445799	8.41635213
hsa-miR-98-5p	0.0389	0.98505182	16.6540926	4.53330794	5.17059203
hsa-miR-137	0.05636	0.98505182	13.3499887	4.51787726	4.59799521
hsa-miR-133b	0.0564	0.98505182	15.3491974	4.52257407	5.59612575
hsa-miR-18a-3p	0.07115	0.98505182	23.9183393	4.50571363	11.9202467
hsa-miR-205-5p	0.08866	0.98505182	17.0025174	4.50426919	8.86989056
hsa-miR-101-3p	0.10302	0.98505182	12.7058017	4.51439552	6.36819697
hsa-miR-193a-5p	0.1031	0.98505182	12.3077184	4.50937907	6.13770017
hsa-miR-24-3p	0.11365	0.98505182	13.0923126	4.5139961	7.29452098
hsa-miR-181a-5p	0.1179	0.98505182	23.8698653	4.40624535	19.5374492
hsa-miR-132-3p	0.1385	0.98505182	12.1493961	4.54630319	7.89728881
hsa-miR-224-5p	0.14558	0.98505182	10.2945438	4.49357096	6.42472759
hsa-miR-21-5p	0.15742	0.98505182	8.58144692	4.49706378	5.66754867
hsa-miR-28-5p	0.15758	0.98505182	11.2259914	4.52464976	8.44612677
hsa-miR-7-5p	0.15811	0.98505182	11.7070671	4.50713123	8.90995778
hsa-miR-16-5p	0.17601	0.98505182	8.31303881	4.50464691	5.60468666
hsa-miR-92a-3p	0.18408	0.98505182	9.34783111	4.48151291	7.97082183
hsa-miR-193b-3p	0.18545	0.98505182	8.81869153	4.50702346	7.23025225
hsa-miR-26a-5p	0.19287	0.98505182	9.4826688	4.50018905	8.67786102
hsa-miR-519d-3p	0.19562	0.98505182	7.56744001	4.52266331	5.47004671
hsa-let-7e-5p	0.20831	0.98505182	8.48908383	4.51166754	7.82976127
hsa-miR-372-3p	0.2104	0.98505182	6.79571811	4.49857898	4.69853952
hsa-miR-34a-5p	0.23714	0.98505182	6.60715452	4.51615958	6.00883201
hsa-miR-365a-3p	0.25319	0.98505182	6.3439022	4.50659184	5.97060025
hsa-miR-30a-3p	0.26651	0.98505182	5.27462758	4.53522683	3.57020641
hsa-miR-30c-5p	0.26767	0.98505182	6.18715894	4.52790403	7.70463745
hsa-miR-449a	0.29846	0.98505182	5.64256056	4.48086726	7.72106827
hsa-miR-30a-5p	0.31634	0.98505182	4.82777977	4.50524126	5.24496524
hsa-miR-200c-3p	0.34573	0.98505182	4.26980647	4.5027342	5.69733725
hsa-miR-133a-3p	0.35382	0.98505182	4.27903854	4.51199273	5.06191558
hsa-miR-222-3p	0.36271	0.98505182	3.67951847	4.51824471	16.2307295
hsa-let-7g-5p	0.37	0.98505182	3.49452226	4.47374033	11.0734926
hsa-let-7c-5p	0.37386	0.98505182	3.84266669	4.48377641	6.95041545
hsa-miR-302c-3p	0.37905	0.98505182	4.0772112	4.51927249	3.85239706
hsa-miR-195-5p	0.38072	0.98505182	3.63560685	4.489536	7.18263566
hsa-miR-363-3p	0.38467	0.98505182	4.02311555	4.54762788	4.69125766
hsa-miR-221-3p	0.39196	0.98505182	2.85757274	4.50764597	12.1911146
hsa-miR-99a-5p	0.39793	0.98505182	3.3775303	4.47283079	7.29379009
hsa-miR-145-5p	0.40891	0.98505182	3.4243923	4.49580046	5.81348158
hsa-miR-34c-5p	0.4141	0.98505182	3.36698897	4.54690109	6.32638513
hsa-miR-181d-5p	0.41431	0.98505182	3.54486409	4.53380472	5.51454428
hsa-miR-20a-5p	0.41968	0.98505182	3.23483087	4.49298228	5.90691991
hsa-miR-22-3p	0.43482	0.98505182	3.19722166	4.5398016	5.74691583
hsa-miR-503-5p	0.45402	0.98505182	2.56086371	4.48875388	6.89341641
hsa-miR-216b-5p	0.45601	0.98505182	2.66345823	4.52076712	7.0463527
hsa-let-7b-5p	0.45912	0.98505182	2.5748008	4.50137391	6.75922459
hsa-miR-155-5p	0.46007	0.98505182	2.06614701	4.47848565	8.17281854
hsa-miR-1	0.4652	0.98505182	2.18263419	4.52521414	8.11280445
hsa-miR-16-1-3p	0.48069	0.98505182	2.65121844	4.50299922	5.76217578
hsa-miR-138-5p	0.48301	0.98505182	2.65190909	4.51283505	5.60766317
hsa-miR-125b-5p	0.48691	0.98505182	2.57643432	4.49505509	5.65629667
hsa-miR-569	0.48732	0.98505182	1.17942718	4.50820728	10.2387061
hsa-miR-122-5p	0.49183	0.98505182	2.72702854	4.53050119	5.11391314
hsa-miR-542-5p	0.5067	0.98505182	2.60284159	4.51472505	5.03555466

hsa-miR-126-3p	0.52899	0.98505182	1.21734698	4.51133374	7.81910245
hsa-miR-296-5p	0.53626	0.98505182	1.40790613	4.52063698	8.0931866
hsa-miR-10b-5p	0.54084	0.98505182	2.39647562	4.51384718	5.69564599
hsa-miR-146a-5p	0.56594	0.98505182	0.07540695	4.4805105	9.43367635
hsa-miR-106a-5p	0.5679	0.98505182	1.6888129	4.49225405	6.27649344
hsa-miR-107	0.57151	0.98505182	1.10836889	4.5234359	7.27372027
hsa-miR-18a-5p	0.5789	0.98505182	1.11462583	4.49550866	7.32479024
hsa-miR-99b-5p	0.59453	0.98505182	0.0047387	4.52525219	9.6499216
hsa-miR-520b	0.59644	0.98505182	0.09713506	4.53824165	9.41342548
hsa-miR-17-5p	0.60112	0.98505182	1.63500839	4.49180458	5.97244241
hsa-miR-29b-3p	0.60815	0.98505182	0.02176796	4.50963972	9.41295206
hsa-miR-217	0.61106	0.98505182	1.6956487	4.50578184	6.4974647
hsa-miR-302a-3p	0.62402	0.98505182	0.34488935	4.50235452	7.86795753
hsa-miR-204-5p	0.63769	0.98505182	0.26731073	4.51292654	8.1258885
hsa-miR-34b-5p	0.64117	0.98505182	1.27176028	4.49927621	6.46188044
hsa-miR-203a	0.64667	0.98505182	0.31012059	4.47292915	8.99378287
hsa-let-7a-5p	0.65555	0.98505182	1.15537852	4.52741001	5.99609011
hsa-miR-522-3p	0.66454	0.98505182	0.07169003	4.51650712	8.89792546
hsa-miR-194-5p	0.68125	0.98505182	1.47214081	4.51120364	4.99175674
hsa-miR-494-3p	0.68399	0.98505182	0.44580026	4.46493785	8.06335499
hsa-miR-106b-5p	0.69651	0.98505182	0.83543575	4.50140683	6.27353306
hsa-miR-622	0.71879	0.98505182	0.55477036	4.49118	6.65496329
hsa-miR-26b-5p	0.72059	0.98505182	1.75163091	4.52595236	4.60510112
hsa-miR-424-5p	0.72381	0.98505182	0.00011211	4.47255023	7.33065884
hsa-miR-93-5p	0.72762	0.98505182	0.00548456	4.50962782	7.14699454
hsa-miR-214-3p	0.76607	0.98505182	0.9178704	4.50300178	5.74656238
hsa-miR-335-5p	0.76641	0.98505182	0.6332284	4.51231715	6.01895117
hsa-miR-31-5p	0.76859	0.98505182	0.00553574	4.49535386	6.96193014
hsa-miR-125a-5p	0.77768	0.98505182	0.43447145	4.52204745	6.31059781
hsa-miR-29a-3p	0.7852	0.98505182	0.05298802	4.50133875	6.79256432
hsa-miR-301a-3p	0.79899	0.98505182	0.52370331	4.52175318	5.94260878
hsa-miR-199a-3p	0.80707	0.98505182	0.73326793	4.53302965	5.4851674
hsa-miR-124-3p	0.82747	0.98505182	1.4508853	4.51688965	4.29382373
hsa-miR-373-3p	0.82836	0.98505182	0.42022057	4.50355979	5.66173593
hsa-miR-29c-3p	0.83266	0.98505182	0.10253288	4.49939308	6.10552245
hsa-miR-302b-3p	0.84016	0.98505182	0.00253399	4.48899151	6.20959488
hsa-miR-433-3p	0.84493	0.98505182	0.88112286	4.51561069	4.91215872
hsa-miR-30d-5p	0.84562	0.98505182	0.04598701	4.49586403	6.11331744
hsa-miR-193a-3p	0.8486	0.98505182	0.1215158	4.50599737	6.00358832
hsa-miR-34b-3p	0.86855	0.98505182	0.21025861	4.51257068	5.66147786
hsa-miR-223-3p	0.87205	0.98505182	0.4150613	4.49857954	5.33357041
hsa-miR-483-5p	0.88279	0.98505182	1.60E-06	4.50691371	5.79214501
hsa-miR-96-5p	0.88521	0.98505182	0.75892399	4.50968798	4.80325616
hsa-miR-378a-5p	0.88574	0.98505182	0.0266626	4.50181012	5.68374493
hsa-miR-199b-3p	0.88952	0.98505182	0.05558671	4.54375284	5.71967312
hsa-miR-19b-3p	0.90345	0.98505182	0.0049691	4.51766568	5.62401998
hsa-miR-100-5p	0.90955	0.98505182	0.20607788	4.49459777	5.38429649
hsa-miR-19a-3p	0.91967	0.98505182	0.05367477	4.51703044	5.46543203
hsa-miR-128-3p	0.92713	0.98505182	0.0809657	4.53266378	5.32931191
hsa-miR-30b-5p	0.93956	0.98505182	0.71601019	4.51867667	4.39664405
hsa-miR-483-3p	0.94264	0.98505182	0.3090737	4.50196369	4.57962017
hsa-miR-143-3p	0.94325	0.98505182	0.23873255	4.52987003	4.85593598
hsa-miR-519a-3p	0.95388	0.98505182	0.28406977	4.53133289	4.49141695
hsa-miR-146b-5p	0.9589	0.98505182	0.31489614	4.52871511	4.62201882
hsa-miR-378a-3p	0.97834	0.99149	0.79982776	4.52956256	3.66374618
hsa-miR-182-5p	0.98984	0.99149	0.22980887	4.5312861	4.05026966
hsa-miR-451a	0.99149	0.99149	0.15695454	4.50764582	3.84715184
